# Assessment of coronavirus disease 2019 mRNA vaccine response in kidney transplant recipients

**DOI:** 10.1371/journal.pone.0359640

**Published:** 2026-11-04

**Authors:** Mohamad M. Alkadi, Essa A. Abuhelaiqa, Rasha M. Abdul Rahman, Ali B. Mahgoub, Abdullah I. Hamad, Peter V. Coyle, Zahir M. Tag, Laith J. Abu Raddad, Hassan A. Al-Malki

**Affiliations:** 1 Department of Medicine, Division of Nephrology, Hamad Medical Corporation, Doha, Qatar; 2 Department of Laboratory Medicine and Pathology, Hamad Medical Corporation, Doha, Qatar; 3 Infectious Disease Epidemiology Group, Weill Cornell Medicine–Qatar, Cornell University, Doha, Qatar; Brigham and Women’s Hospital, UNITED STATES OF AMERICA

## Abstract

Kidney transplant recipients are vulnerable to developing severe coronavirus disease 2019 (COVID-19) infection. Although mRNA vaccines have significantly improved outcomes, seropositivity rates remain variable in immunosuppressed patients. This study aimed to evaluate the antibody response to COVID-19 mRNA vaccines, identify factors affecting immunogenicity, and examine the association between antibody titers and subsequent SARS-CoV-2 infection and COVID-19-related hospitalization among kidney transplant recipients. We retrospectively studied 372 adult kidney transplant recipients who received two doses of COVID-19 mRNA vaccine after transplantation and had anti-SARS‑CoV‑2 spike protein receptor-binding domain antibodies measured ≥ 14 days after the second dose and prior to receiving a third dose. We categorized patients based on previous COVID-19 infection, post-vaccination antibody status (seropositive or seronegative), and antibody titers (low or high). Factors associated with poor vaccine response (seronegativity) and with high antibody titers (antibody titer ≥ 121 U/mL) were determined using multivariable regression models. The proportion of patients with subsequent COVID-19 infection was higher in the seronegative group than in the seropositive or previous infection groups (36% vs. 26% vs. 13%, respectively; P = 0.02). Multivariable regression analysis revealed higher odds of poor response to the mRNA vaccine among patients aged > 52 years compared with those aged ≤ 52 years (aOR = 1.94; 95% CI: 1.12–3.36; P = 0.018). Lower odds were observed among male patients (aOR = 0.43; 95% CI: 0.24–0.77; P = 0.004) and those taking azathioprine (aOR = 0.26; 95% CI: 0.07–0.91; P = 0.035). The proportion of patients with subsequent COVID-19 was also higher in the low-antibody group than in the high-antibody group (33% vs. 20%; P = 0.02). Multivariable logistic regression analysis showed lower odds of high SARS-CoV-2 antibody titers among patients aged > 51 years compared with those aged ≤ 51 years (aOR = 0.43; 95% CI: 0.24–0.75; P = 0.003). Higher odds were observed among those of African race (aOR = 2.82; 95% CI: 1.11–7.16; P = 0.03) and South Asian race (aOR = 2.27; 95% CI: 1.08–4.75; P = 0.03) compared with Middle Eastern recipients. Understanding the immunologic response to the SARS-CoV-2 vaccine in kidney transplant recipients is essential for preventing potentially life-threatening infection. Furthermore, identifying transplant recipients with a low vaccine-induced antibody response may help guide the development of individualized immunization strategies.

## 1. Introduction

Since its emergence at the end of 2019, coronavirus disease 2019 (COVID-19) has affected millions worldwide, creating a significant public health burden [1]. Kidney transplant recipients are particularly susceptible to developing severe COVID-19 infection due to their chronic immunosuppression, which compromises their immune response to pathogens and vaccination alike [2,3]. Although the COVID-19 pandemic accelerated the development of messenger ribonucleic acid (mRNA) vaccines, which have proven effective in preventing severe disease in the general population, their effectiveness in kidney transplant recipients has been less than optimal [4–8]. This suboptimal response leads to increased risks of severe illness, hospitalization, and mortality, and may also adversely affect graft survival. Therefore, evaluating the response to COVID-19 mRNA vaccines, including the kinetics and magnitude of the antibody response, is crucial in kidney transplant recipients.

The causative agent of COVID-19 is severe acute respiratory syndrome coronavirus 2 (SARS-CoV-2), an enveloped single-stranded RNA Betacoronavirus [9]. The virus enters host cells by binding the receptor-binding domain (RBD) of its S1 subunit to the angiotensin-converting enzyme 2. Infection with SARS-CoV-2 elicits an antibody response against the spike protein. As SARS-CoV-2 has undergone numerous mutations, neutralizing antibodies against prior strains have been shown to be less effective against newer variants, such as Delta and Omicron, in non-immunocompromised individuals [10]. However, this effect has not been well evaluated in kidney transplant recipients, whose compromised immune systems may further limit their ability to mount and sustain protective responses against evolving viral strains [11].

In this study, we aimed to evaluate the antibody response rate to COVID-19 mRNA vaccines among kidney transplant recipients, identify factors affecting immunogenicity, and examine the association between antibody titers and subsequent SARS-CoV-2 infection and COVID-19-related hospitalization in this high-risk patient population.

## 2. Materials and methods

### 2.1. Study population and design

This was a retrospective cohort study of adult kidney transplant recipients who received two doses of COVID-19 mRNA vaccine after transplantation and had anti‑SARS‑CoV‑2 S protein RBD antibodies measured at least 14 days after the second dose and before receiving a third booster dose. These criteria were applied before initiation of anti‑SARS‑CoV‑2 antibody testing, which was performed from 1 June to 30 September 2021. Owing to limited availability of the anti‑SARS‑CoV‑2 immunoassay, only 372 of the 577 eligible patients underwent antibody testing and were subsequently followed until 1 February 2022.

In Qatar, COVID-19 vaccines were administered exclusively at governmental health centers, and mRNA COVID-19 vaccines were the only vaccines authorized and used. The COVID-19 vaccine booster program was launched in Qatar on 24 August 2021.

We categorized tested patients based on previous COVID-19 infection, post-vaccination antibody status (seropositive or seronegative), and antibody titers (low or high based on the median level of antibodies). The study design is shown in Fig 1.

**Fig 1:**
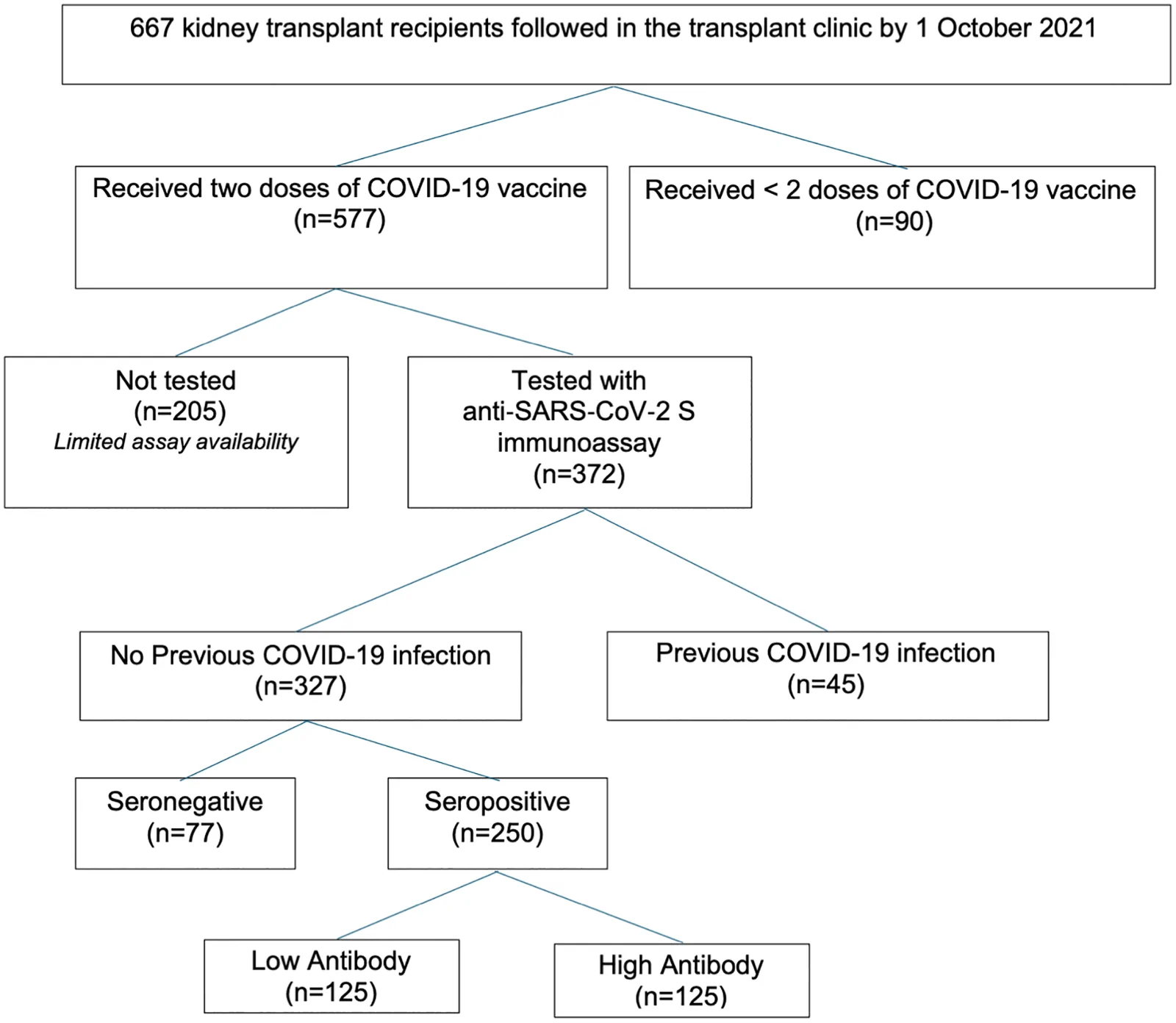
Study flow diagram of kidney transplant recipients who received two doses of COVID-19 mRNA vaccine and underwent anti‑SARS‑CoV‑2 S protein antibody testing. Patients were categorized according to prior COVID-19 infection, post-vaccination antibody status (seropositive vs. seronegative), and antibody titers (low vs. high, based on the median).

### 2.2. Anti-SARS-CoV-2 antibody measurement

Anti-SARS-CoV-2 S protein RBD antibodies, including IgG, were quantitatively determined using Elecsys® anti‑SARS‑CoV‑2 S immunoassay. The measuring range of this assay is 0.40‑250 U/mL. For values > 250 U/mL, a value of 250 U/mL was assigned in the analysis. The manufacturer-defined threshold for a positive result using the Elecsys® anti–SARS-CoV-2 S immunoassay is 0.80 U/mL. Thus, patients were considered seropositive if their anti-SARS-CoV-2 S protein RBD antibody titer was ≥ 0.80 U/mL, and seronegative if below this threshold.

Poor response was defined as the failure to achieve seropositivity at least 14 days after receiving two doses of the vaccine (antibody titer < 0.80 U/mL). Since a previous COVID-19 infection can result in a positive Elecsys® anti‑SARS‑CoV‑2 S immunoassay, patients with prior COVID-19 infection were analyzed separately as a distinct group. The median antibody titer of the seropositive group was used as the cutoff to divide patients into “low” and “high” antibody titer subgroups. This cutoff served as an internal, data-driven stratification for analytical purposes within this study.

### 2.3. Data collection

All clinical and laboratory data were collected using a nationwide electronic medical record system (Cerner, North Kansas City, MO, USA). This system includes all clinic visits, emergency department visits, hospitalizations, imaging, and laboratory tests conducted at any government hospital or primary healthcare center across Qatar. Vaccine type and administration dates are also recorded in Cerner. All COVID-19 infections included in the study were confirmed by polymerase chain reaction (PCR) testing. During the pre-Omicron period, COVID-19 diagnosis was based exclusively on PCR testing. However, during the Omicron-predominant period, rapid antigen testing was also used, with all positive antigen test results confirmed by PCR.

This study was approved by our local institutional review board (MRC-01-21-841). The requirement for informed consent was waived, as the study was a retrospective review of medical records. All authors had access to information that could identify individual participants during data collection.

### 2.4. Statistical analysis

Associations with poor response to the COVID-19 mRNA vaccine and with high SARS-CoV-2 antibody titers were first examined using univariable logistic regression models. Continuous variables were recoded and dichotomized at the median, such that patients scoring at or below the median were assigned to the lower group, and those scoring above the median to the higher group.

For comparisons across groups, categorical variables were analyzed using the chi-square test or Fisher’s exact test, as appropriate. Continuous variables with an approximately normal distribution were compared using one-way analysis of variance (ANOVA), whereas variables with a non-normal or markedly skewed distribution were compared using the Kruskal-Wallis test. Variables with a P value ≤ 0.20 in univariable analyses were subsequently entered into a multivariable logistic regression model. Statistical significance in the multivariable model was determined at a P value < 0.05. This approach was chosen because both outcomes (poor response to the COVID-19 mRNA vaccine and high SARS-CoV-2 antibody titers) were binary variables derived from a single antibody measurement obtained following the second vaccine dose, and the primary objective was to assess associations with antibody response. Effect estimates were reported as odds ratios (ORs) and adjusted odds ratios (aORs), with corresponding 95% confidence intervals (CIs).

Time to COVID-19 infection was analyzed using Kaplan–Meier survival analysis, with the date of the anti-SARS-CoV-2 S immunoassay test as time zero and subsequent COVID-19 infection as the event. Participants who did not develop COVID-19 infection were censored on February 1, 2022. No participants were censored because of death, loss to follow-up, or receipt of a third vaccine dose. Kaplan–Meier curves for the four groups were compared using an overall log-rank test. No pairwise comparisons were performed.

Although post-vaccination COVID-19 infections during follow-up were recorded, Cox proportional hazards regression was not performed because this would have introduced several methodological complexities beyond the scope of the present study, including the need to incorporate the third vaccine dose as a time-varying exposure.

All statistical analyses were performed using Stata/SE version 18.0 (Stata Corporation, College Station, TX, USA).

## 3. Results

### 3.1. Baseline characteristics and humoral response to COVID-19 mRNA vaccination

We included 372 of 577 eligible adult kidney transplant recipients (64%) who underwent testing for anti-SARS‑CoV‑2 S protein RBD antibodies after receiving two doses of the COVID-19 mRNA vaccine and before receiving a third vaccine dose, owing to limited immunoassay availability. Baseline characteristics were similar between the tested and untested groups, with no significant differences in age, sex, race, or year of transplant (S1 Table).

Forty-five patients had a previous COVID-19 infection before anti‑SARS‑CoV‑2 S immunoassay testing. Of these, 21 patients had COVID-19 infection before vaccination, while 24 patients had infection after vaccination (6 patients developed infection after the first vaccine dose but before the second dose, and 18 patients developed infection after the second dose). The median time from infection to antibody testing was 381 days (IQR 289–421) for infections before vaccination, 112 days (IQR 99–131) for infections after the first dose, and 88.5 days (IQR 65.5–110.8) for infections after the second dose. The interval differed significantly across groups (P < 0.001). Despite these differences in timing, comparison of antibody levels across the three groups (infection before vaccination, infection after the first dose but before the second dose, and infection after the second dose) showed no statistically significant difference in antibody titers, with median antibody level of 250 U/mL in all three groups (P = 0.54). In addition, Spearman correlation analysis demonstrated no significant association between the time from infection to antibody testing and antibody titers (ρ = −0.064, P = 0.68).

Of the remaining 327 patients with no documented previous infection, 250 (76%) were seropositive. Among them, 125 had low antibody titers (anti-SARS‑CoV‑2 S protein RBD antibody titer < 121 U/mL) and 125 had high antibody titers (anti-SARS‑CoV‑2 S protein RBD antibody titer ≥ 121 U/mL), as shown in Fig 1.

Most kidney transplant recipients received the Pfizer-BioNTech mRNA vaccine (96%), while the remaining patients (4%) received the Moderna vaccine. Seropositivity after two doses of the COVID-19 mRNA vaccine was more frequent in patients with previous COVID-19 infection than in those without previous infection (96% vs. 76%; P = 0.002). In addition, the median anti-SARS-CoV-2 S protein RBD antibody titer in the previous COVID-19 infection group was 250 U/mL, corresponding to the assay’s upper measurement limit. There was no significant difference in the number of days between the second vaccine dose and the anti‑SARS‑CoV‑2 S immunoassay across the groups (P = 0.23).

### 3.2. Post-vaccination COVID-19 infections and clinical outcomes

The proportion of patients with subsequent COVID-19 infection after anti‑SARS‑CoV‑2 S immunoassay testing was 13%, 26%, and 36% in the previous COVID-19 infection, seropositive and seronegative groups, respectively (P = 0.02). The median time between anti‑SARS‑CoV‑2 S immunoassay testing and post-testing COVID-19 infection was similar among the groups (P = 0.50).

During the follow-up period after anti‑SARS‑CoV‑2 S immunoassay testing, 74% of patients received a third COVID-19 mRNA vaccine (n = 275), with no difference across groups (P = 0.65). Among patients with previous infection, 6 developed COVID-19 infection (3 after the third vaccine dose and 3 who did not receive the third dose). Among seropositive patients, 66 developed infection (1 before the third dose, 38 after the third dose, and 27 who did not receive the third dose). Among seronegative patients, 28 developed infection (22 after the third dose and 6 who did not receive the third dose).

The time between the third vaccine dose and subsequent COVID-19 infection differed significantly among the groups (P = 0.04).

There was no difference in hospitalization rates among the groups (P = 0.90), and only one patient in the seronegative group required intensive care unit admission. Data are summarized in Table 1.

**Table 1:** Baseline characteristics, humoral response and clinical outcomes in kidney transplant recipients according to previous COVID-19 infection and post-vaccination antibody status.

	Previous COVID-19 Infection (n = 45)	No Previous Infection (n = 327)		P value*
		Seropositive (n = 250)	Seronegative (n = 77)	
**Age, years, median (IQR)**	55 (42-64)	51 (39 - 61)	55 (44- 63)	0.06
**Male sex, n (%)**	32 (71)	188 (75)	48 (62)	0.09
**Race, n (%)** **Middle East** **South Asia** **East Asia** **Africa** **Others**	29 (64) 9 (20) 1 (2) 3 (7) 3 (7)	168 (67) 41 (16) 16 (6) 24 (10) 1 (0.4)	54 (70) 8 (10) 7 (9) 7 (9) 1 (1)	0.05
**Type of mRNA vaccine, n (%)** **Pfizer-BioNTech** **Moderna**	43 (96) 2 (4)	240 (96) 10 (4)	73 (95) 4 (5)	0.90
**SARS-CoV-2 antibody titer, median (IQR)**	250 (250−250)	121 (25-250)	0.4 (0.4-0.4)	< 0.0001
**Days between the second vaccine dose and immunoassay, median (IQR)**	125 (93-153)	137 (108-162)	137 (99-159)	0.23
**Third dose of COVID-19 vaccine, n (%)**	31 (69)	185 (74)	59 (77)	0.65
**COVID-19 infection, n (%)**	6 (13)	66 (26)	28 (36)	0.02
**Days between immunoassay and COVID-19 infection, median (IQR)**	164.5 (139-188)	179 (149-199)	178 (160-197)	0.50
**Days between the third vaccine dose and COVID-19 infection, median (IQR)**	118 (96-119)	128 (103-133)	132 (127-141)	0.04
**Hospitalization, n (%)**	2 (4)	8 (3)	3 (4)	0.90
**ICU admission, n (%)**	0	0	1 (1)	0.15

* P values for categorical variables were calculated using the chi-square test or Fisher’s exact test, as appropriate. Continuous variables with an approximately normal distribution were compared using one-way analysis of variance (ANOVA), whereas variables with a non-normal or markedly skewed distribution were compared using the Kruskal–Wallis test. Specifically, ANOVA was used for age, while the Kruskal–Wallis test was used for SARS-CoV-2 antibody titer, days between the second vaccine dose and immunoassay, days between immunoassay and COVID-19 infection, and days between the third vaccine dose and COVID-19 infection.

### 3.3. Seronegative versus seropositive post COVID-19 vaccine groups

We categorized patients with no previous COVID-19 infection into two groups: seronegative (n = 77) and seropositive (n = 250). Female sex was more common in the seronegative group compared with the seropositive group (38% versus 25%; P = 0.03). Taking azathioprine was more common in the seropositive group compared with the seronegative group (13% versus 4%; P = 0.03). The median age was higher in the seronegative group compared with the seropositive group, but this difference was not statistically significant (55 versus 51 years; P = 0.06). Both groups had a similar number of days between the first and second vaccine doses and between the second dose and antibody titer measurement. The baseline characteristics of both groups are summarized in Table 2.

**Table 2:** Baseline characteristics of seronegative and seropositive groups.

Variable	Seronegative (n = 77)	Seropositive (n = 250)	P value
**Age, years, median (IQR)**	55 (44- 63)	51 (39 - 61)	0.06
**Male sex, n (%)**	48 (62)	188 (75)	0.03
**Race, n (%)** **Middle East** **South Asia** **East Asia** **Africa** **Others**	54 (70) 8 (10) 7 (9) 7 (9) 1 (1)	168 (67) 41 (16) 16 (6) 24 (10) 1 (0.4)	0.58
**History of diabetes, n (%)**	43 (56)	122 (49)	0.27
**Immunosuppression, n (%)** **Tacrolimus** **Cyclosporine** **Mycophenolate Mofetil** **Azathioprine** **Sirolimus** **Prednisolone**	67 (87) 7 (9) 65 (84) 3 (4) 4 (5) 68 (88)	197 (79) 41 (16) 202 (81) 32 (13) 8 (3) 221 (88)	0.11 0.11 0.47 0.03 0.49 0.98
**CNI level before first dose of vaccine, ng/mL, median (IQR)** **Tacrolimus** **Cyclosporine**	6.3 (5.5-7.6) 77 (65-145)	6.4 (5.5-7.4) 94 (84-118)	0.65 0.83
**Serum creatinine before the first vaccine dose, μmol/L, median (IQR)**	106 (86 - 133)	100 (82 - 122)	0.07
**Months from transplant to first vaccine dose, median (IQR)**	72 (29- 137)	86 (53-158)	0.52
**Days between first and second vaccine doses, median (IQR)**	21 (21 −21)	21 (21 − 21)	0.65
**Days between second vaccine dose and antibody test, median (IQR)**	137 (98–161)	137 (108 −162)	0.19

Factors associated with a poor response to the COVID-19 mRNA vaccine were determined using univariable and multivariable regression models. The multivariable regression analysis revealed higher odds of poor response to the COVID-19 mRNA vaccine among patients aged > 52 years compared with those aged ≤52 years (aOR = 1.94; 95% CI: 1.12–3.36; P = 0.018). Lower odds were observed among male patients (aOR = 0.43; 95% CI: 0.24–0.77; P = 0.004) and those taking azathioprine (aOR = 0.26; 95% CI: 0.07–0.91; P = 0.035). No significant associations with poor vaccine response were found for race, diabetes, use of tacrolimus, cyclosporine, mycophenolate mofetil, sirolimus, or prednisolone, vaccine type, serum creatinine before the first vaccine dose, time from transplantation to the first vaccine dose, days between the first and second vaccine doses, or days between the second vaccine dose and antibody testing. The results of the univariable and multivariable regression analyses are summarized in Table 3.

**Table 3:** Factors associated with poor response to the COVID-19 mRNA vaccine.

Characteristics	Univariable regression analysis			Multivariable regression analysis	
	OR (95% CI)	P value	LR test P value^*^	aOR (95% CI)	P value^†^
**I. Demographic and clinical characteristics**					
Age					
Younger than the median	1.00		0.100	1.00	
Older than the median	1.54 (0.92-2.58)	0.100		1.94 (1.12-3.36)	0.018
Sex					
Female	1.00		0.031	1.00	
Male	0.55 (0.32-0.94)	0.029		0.43 (0.24-0.77)	0.004
Race					
Middle East	1.00		0.465	--	--
Africa	0.91 (0.37-2.22)	0.832		--	--
South Asia	0.61 (0.27-1.37)	0.231		--	--
Other regions^‡^	1.46 (0.60-3.58)	0.404		--	--
Diabetes					
No	1.00		0.293	--	--
Yes	1.32 (0.79-2.20)	0.294		--	--
**II. Immunosuppressive medication use**					
Tacrolimus					
No	1.00		0.098	1.00	
Yes	1.80 (0.87-3.74)	0.114		0.89 (0.24-3.21)	0.853
Cyclosporine					
No	1.00		0.098	1.00	
Yes	0.51 (0.22-1.19)	0.119		0.56 (0.13-2.44)	0.441
Mycophenolate Mofetil					
No	1.00		0.467	--	--
Yes	1.29 (0.64-2.57)	0.474		--	--
Azathioprine					
No	1.00		0.015	1.00	
Yes	0.28 (0.08-0.93)	0.038		0.26 (0.07-0.91)	0.035
Sirolimus					
No	1.00		0.434	--	--
Yes	1.66 (0.49-5.66)	0.420		--	--
Prednisolone					
No	1.00		0.983	--	--
Yes	0.99 (0.45-2.20)	0.983		--	--
**III. CNI level before first dose of vaccine**					
Tacrolimus					
Lower than the median	1.00		0.358	--	--
Higher than the median	0.77 (0.44-1.35)	0.359		--	--
Cyclosporine					
Lower than the median	1.00		0.409	--	--
Higher than the median	0.48 (0.08-2.90)	0.420		--	--
**IV. Vaccine factors**					
Vaccine type					
Moderna	1.00		0.658	--	--
Pfizer	0.76 (0.23-2.50)	0.652		--	--
Serum creatinine before the first vaccine dose					
Lower than the median	1.00		0.514	--	--
Higher than the median	1.19 (0.71-1.98)	0.515		--	--
Months from transplant to first vaccine dose					
Earlier than the median	1.00		0.054	1.00	
Later than the median	0.60 (0.36-1.01)	0.056		0.72 (0.41-1.28)	0.264
Days between first and second vaccine doses					
Earlier than the median	1.00		0.533	--	--
Later than the median	0.80 (0.39-1.64)	0.539		--	--
Days between second vaccine dose and antibody test					
Earlier than the median	1.00		0.982	--	--
Later than the median	1.01 (0.60-1.68)	0.982		--	--

Abbreviations: aOR, adjusted odds ratio; CI, confidence interval; CNI, calcineurin inhibitor; COVID-19, coronavirus disease 2019; LR, likelihood ratio; mRNA, messenger ribonucleic acid; OR, odds ratio.

* Covariates with P value ≤ 0.20 in the univariable analysis were included in the multivariable analysis.

† Covariates with P value < 0.05 in the multivariable analysis were considered as showing statistically significant evidence for an association with poor response to the COVID-19 mRNA vaccine.

‡ The ‘Others’ category was combined with East Asia due to having only two observations.

### 3.4. Low versus high anti-SARS-CoV-2 antibody groups

We divided seropositive kidney transplant recipients into low- and high-antibody groups based on the median anti-SARS-CoV-2 S protein RBD antibody titer value (low-antibody group: titer < 121 U/mL, n = 125; high-antibody group: titer ≥ 121 U/mL, n = 125). The median age was higher in the low-antibody group (56 versus 48 years; P < 0.0001). In addition, a history of diabetes was more frequent in the low-antibody group (55% versus 42%; P = 0.04), and the median number of days between the second vaccine dose and antibody testing was also higher in the low-antibody group (142 versus 132 days; P = 0.002). The baseline characteristics of the low- and high-antibody groups are summarized in Table 4.

**Table 4:** Baseline characteristics and outcomes of seropositive kidney transplant recipients stratified by low versus high anti-SARS-CoV-2 antibody titers.

Variable	Low Antibody (n = 125)	High Antibody (n = 125)	P value
**Months from transplant to first vaccine dose, median (IQR)**	86.8 (53.5-162.9)	86.9 (55.5 −146.0)	0.51
**Age, median (IQR)**	56 (43-64)	48 (36-57)	< 0.0001
**Male sex, n (%)**	94 (75)	94 (75)	1
**Race, n (%)** **Middle East** **South Asia** **East Asia** **Africa** **Others**	96 (77) 15 (12) 5 (4) 8 (6) 1 (1)	72 (58) 26 (21) 11 (9) 16 (13) 0 (0)	0.02
**Serum creatinine prior to 1st dose, μmol/L, median (IQR)**	100 (84 −120)	100 (81 −124)	0.86
**CNI level prior to 1st dose, ng/mL, median (IQR)** **Tacrolimus** **Cyclosporine**	6.6 (5.6-7.6) 93 (82 −123)	6.2 (5.5- 7.3) 100 (85-111)	0.49 0.34
**History of diabetes, n (%)**	69 (55)	53 (42)	0.04
**Days between the second vaccine dose and antibody testing, median (IQR)**	142 (123-168)	132 (100-155)	0.002
**Immunosuppression, n (%):** **Tacrolimus** **Cyclosporine** **Mycophenolate Mofetil** **Azathioprine** **Sirolimus** **Prednisolone**	102 (82) 17 (14) 103 (82) 14 (11) 4 (3) 110 (88)	95 (76) 24 (19) 99 (79) 18 (14) 4 (3) 111 (89)	0.28 0.67 0.41 0.45 1.0 0.84
**COVID-19 infection, n (%)**	41 (33)	25 (20)	0.02
**Hospitalization, n (%)**	7 (6)	1 (1)	0.07
**Third dose of COVID-19 vaccine, n (%)**	98 (78)	87 (70)	0.11
**Days between third dose and COVID-19 infection, median (IQR)**	127 (110-132)	130 (104-136)	0.64
**Days between antibody test and COVID-19 infection, median (IQR)**	178 (151-199)	180 (145-197)	0.51

Factors associated with high antibody titers (≥ 121 U/mL) after mRNA vaccine were determined using univariable and multivariable regression models. The multivariable logistic regression analysis revealed lower odds of high SARS-CoV-2 antibody titers among patients aged > 51 years compared with those aged ≤ 51 years (aOR = 0.43; 95% CI: 0.24–0.75; P = 0.003). Higher odds were observed among patients of African race (aOR = 2.82; 95% CI: 1.11–7.16; P = 0.03) and South Asian race (aOR = 2.27; 95% CI: 1.08–4.75; P = 0.03) compared with those of Middle Eastern race. No significant associations with high SARS-CoV-2 antibody titers were found for sex, diabetes, use of tacrolimus, cyclosporine, mycophenolate mofetil, azathioprine, sirolimus, or prednisolone, vaccine type, serum creatinine before the first vaccine dose, time from transplantation to the first vaccine dose, days between the first and second vaccine doses, or days between the second vaccine dose and antibody testing. The results of the univariable and multivariable regression analyses are summarized in Table 5.

**Table 5:** Factors associated with high anti-SARS-CoV-2 antibody titers.

Characteristics	Univariable regression analysis			Multivariable regression analysis	
	OR (95% CI)	P value	LR test P value^*^	aOR (95% CI)	P value^†^
**I. Demographic and clinical characteristics**					
Age					
Younger than the median	1.00		< 0.001	1.00	
Older than the median	0.40 (0.24-0.67)	< 0.001		0.43 (0.24-0.75)	0.003
Sex					
Female	1.00		1.000	--	--
Male	1.00 (0.56-1.78)	1.000		--	--
Race					
Middle East	1.00		0.014	1.00	
Africa	2.67 (1.08-6.57)	0.033		2.82 (1.11-7.16)	0.030
South Asia	2.31 (1.14-4.68)	0.020		2.27 (1.08-4.75)	0.030
Other regions^‡^	2.44 (0.86-6.92)	0.092		2.57 (0.86-7.74)	0.092
Diabetes					
No	1.00		0.049	1.00	
Yes	0.61 (0.37-1.00)	0.050		0.79 (0.45-1.39)	0.418
**II. Immunosuppressive medication use**					
Tacrolimus					
No	1.00		0.278	--	--
Yes	0.71 (0.39-1.32)	0.280		--	--
Cyclosporine					
No	1.00		0.231	--	--
Yes	1.51 (0.77-2.97)	0.234		--	--
Mycophenolate Mofetil					
No	1.00		0.521	--	--
Yes	0.81 (0.43-1.53)	0.521		--	--
Azathioprine					
No	1.00		0.448	--	--
Yes	1.33 (0.63-2.82)	0.450		--	--
Sirolimus					
No	1.00		1.000	--	--
Yes	1.00 (0.24-4.09)	1.000		--	--
Prednisolone					
No	1.00		0.843	--	--
Yes	1.08 (0.50-2.35)	0.843		--	--
**III. CNI level before first dose of vaccine**					
Tacrolimus					
Lower than the median	1.00		0.138^§^	--	--
Higher than the median	0.66 (0.38-1.15)	0.139		--	--
Cyclosporine					
Lower than the median	1.00		0.035^§^	--	--
Higher than the median	4.13 (1.06-16.10)	0.041		--	--
**IV. Vaccine factors**					
Vaccine type					
Moderna	1.00		0.191	1.00	
Pfizer	0.41 (0.10-1.64)	0.210		0.68 (0.15-2.97)	0.604
Serum creatinine before the first vaccine dose					
Lower than the median	1.00		0.951	--	--
Higher than the median	1.02 (0.62-1.67)	0.950		--	--
Months from transplant to first vaccine dose					
Earlier than the median	1.00		1.000	--	--
Later than the median	1.00 (0.61-1.64)	1.000		--	--
Days between first and second vaccine doses					
Earlier than the median	1.00		0.418	--	--
Later than the median	1.31 (0.68-2.54)	0.419		--	--
Days between second vaccine dose and antibody test					
Earlier than the median	1.00		0.057	1.00	
Later than the median	0.62 (0.37-1.02)	0.058		0.79 (0.46-1.36)	0.403

Abbreviations: aOR, adjusted odds ratio; CI, confidence interval; CNI, calcineurin inhibitor; LR, likelihood ratio; OR, odds ratio; SARS-CoV-2, severe acute respiratory syndrome coronavirus 2.

* Covariates with P value ≤ 0.20 in the univariable analysis were included in the multivariable analysis.

† Covariates with P value < 0.05 in the multivariable analysis were considered as showing statistically significant evidence for an association with high SARS-CoV-2 antibody titers.

‡ The ‘Others’ category was combined with East Asia due to having only one observation.

§ These variables were excluded from the multivariable analysis, as CNI levels were predominantly available for patients who had received the respective immunosuppressants and were largely missing for those who had not, which would have substantially reduced the sample size.

Regarding clinical outcomes, COVID-19 infection was significantly more frequent among recipients with low anti-SARS-CoV-2 antibody titers compared to those with high titers (33% vs. 20%; P = 0.02). More patients in the low-antibody group required hospitalization than in the high-antibody group, although this difference was not statistically significant (6% vs. 1%; P = 0.07) (Table 4).

### 3.5. Time to COVID-19 infection

We compared the time from the anti-SARS‑CoV‑2 S immunoassay test to subsequent COVID-19 infection across the various groups (previous COVID-19 infection, seronegative, seropositive with low-antibody titers, and seropositive with high-antibody titers) using Kaplan-Meier analysis. There was a significant difference in the time from the anti-SARS‑CoV‑2 S immunoassay test to COVID-19 infection among the four groups (overall log-rank test, P = 0.021), as shown in Fig 2.

**Fig 2:**
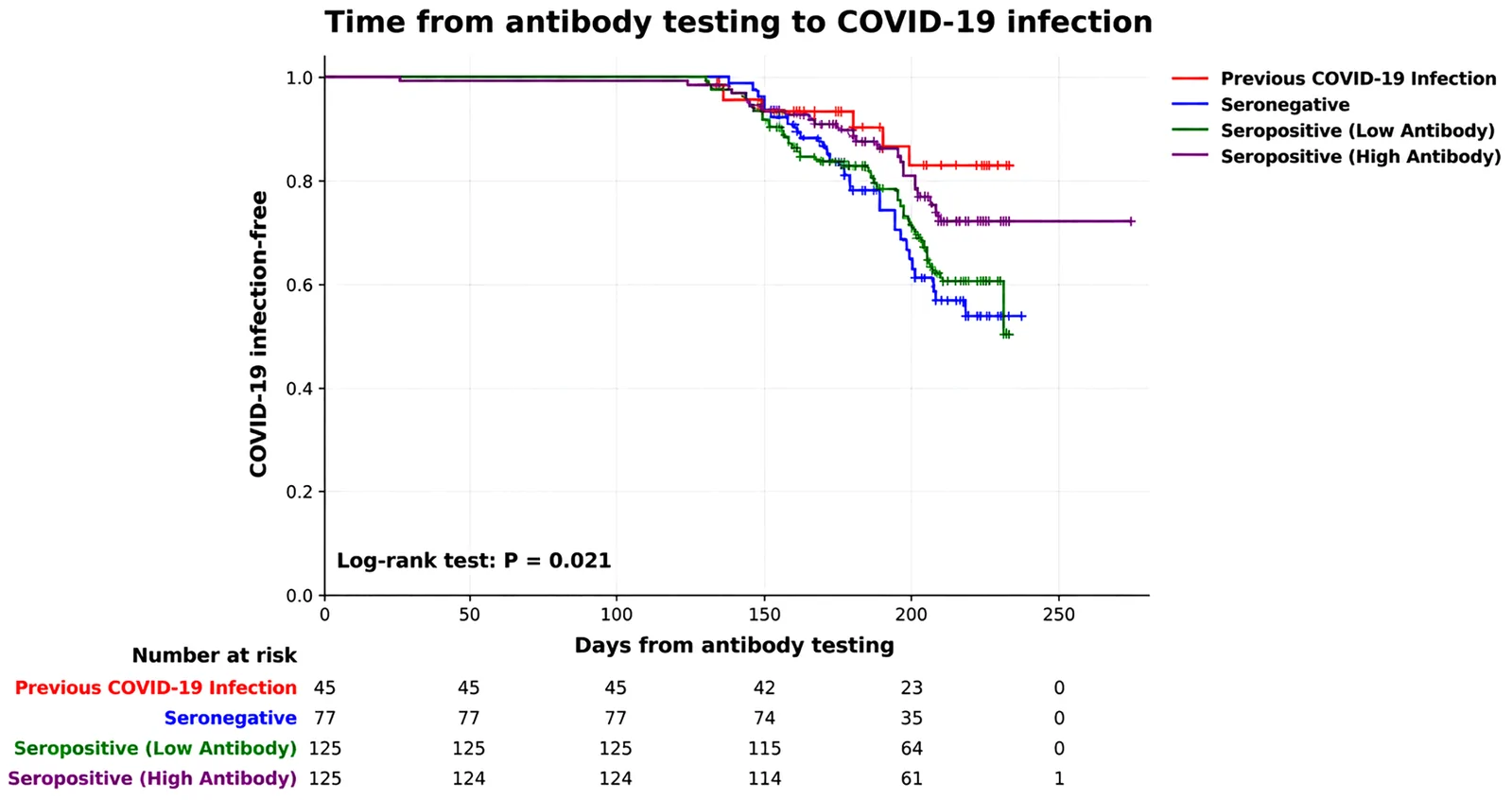
Time from anti-SARS‑CoV‑2 S immunoassay testing to COVID-19 infection in kidney transplant recipients. Patients were stratified by prior COVID-19 infection and antibody response (seronegative, seropositive low, and seropositive high). Kaplan–Meier curves were compared using an overall log-rank test (P = 0.021).

## 4. Discussion

The Omicron SARS-CoV-2 variant (B.1.1.529) was declared by the World Health Organization in November 2021 as a variant of concern due to multiple mutations in the S protein, specifically in the RBD [12–14]. Such mutations have been shown to significantly reduce the neutralizing activity of sera from vaccinated individuals with infection [10,15]. In our study, among vaccinated patients who were seropositive following vaccination, those with high SARS-CoV-2 S protein antibody titers were significantly less likely to develop subsequent COVID-19 infection than those with low antibody titers (20% vs. 33%; P = 0.02), suggesting an association between higher post-mRNA vaccine antibody titers and a lower likelihood of subsequent COVID-19 infection during the Omicron-predominant period.

In our study, we evaluated the humoral immune response to the mRNA vaccine in kidney transplant recipients, and we observed that 76% of transplant recipients had a positive serological response approximately four months after the second dose, which is similar to the response reported by Hovd et al., who measured antibody levels 40 days post-vaccination [16]. However, other studies have reported antibody responses ranging from 4% to 48% in kidney transplant recipients [7,17–19]. Most of these studies measured antibody titers within 15–30 days after vaccination. This difference may be explained by delayed seroconversion, specifically of IgG, in immunocompromised individuals. Kidney transplant recipients have been observed to mount early anti–SARS–CoV–2 IgA and IgM responses, whereas IgG responses were delayed compared with immunocompetent individuals [20]. These findings underscore the critical need for extended monitoring of vaccine responses in kidney transplant recipients, as assessments based on timelines used for the general population may underestimate their true seroconversion rates. Measuring neutralizing antibody levels for SARS-CoV-2 is the gold standard for evaluating vaccine response. However, neutralization testing has largely been limited to research settings because it is labor-intensive [21–23]. Alternatively, anti-SARS-CoV-2 antibody titers measured using enzyme-linked immunosorbent assay (ELISA) may provide a practical alternative, as several studies have demonstrated correlations with neutralizing antibody levels [24–29]. We observed that seropositivity alone was insufficient, as many patients with low SARS-CoV-2 S protein antibody titers still had COVID-19 infection and required hospitalization. Therefore, quantitative assessment of antibody levels may provide additional information beyond seropositivity alone when using commercial assays, as antibody titers may reflect the magnitude of the humoral immune response. This highlights a key implication of our study: simple seropositivity may not fully reflect the magnitude of the antibody response, and quantitative antibody titers may provide additional information when assessing vaccine-induced humoral immunity and its association with subsequent COVID-19 infection in kidney transplant recipients.

Consistent with previous observations, the immunosuppressive regimen influenced the humoral response [30–32]. We found that kidney transplant recipients taking azathioprine were less likely to have a poor response to the mRNA vaccine (aOR = 0.26; 95% CI: 0.07–0.91; P = 0.035). This aligns with studies showing that mycophenolate mofetil significantly attenuates humoral immune responses to SARS-CoV-2 vaccination in transplant recipients by limiting T- and B-cell proliferation [33,34]. Several studies have shown that reducing or suspending mycophenolate mofetil prior to vaccination improved antibody responses [35,36]. However, it is important to note that cessation of mycophenolate mofetil carries risks, including the potential development of donor-specific antibodies (DSA) [37]. Azathioprine, as a weaker antimetabolite, may allow a more favorable balance by permitting humoral immune responses while maintaining protection against DSA development compared with mycophenolate mofetil cessation. Future studies are warranted to comprehensively evaluate azathioprine’s role in vaccine-induced immunity and the associated risk of graft rejection.

Consistent with prior studies, younger patients demonstrated higher seropositivity rates, reflecting their capacity to mount more effective antibody responses than older recipients [30,38–40]. Immunosenescence, an age-related decline in cellular immune function characterized by impaired B- and T-cell interactions and reduced antibody production, has been well described in both the general population and transplant recipients [41,42]. We also observed that male recipients had higher seropositivity rates than females. Stich et al. found that, among kidney transplant recipients, female sex was associated with a 3.11-fold higher risk of lacking a humoral immune response [34]. Conversely, in the general population, women exhibit stronger immune responses to COVID-19 than men, driven by more robust T-cell activation and antibody development [43]. However, in the setting of T cell-targeting immunosuppressants, this sex difference appears reversed, as men exhibit higher levels of innate immune cytokines (IL-8 and IL-18) and greater monocyte activation, which are less affected by immunosuppressive therapy [44].

Measuring antibodies to evaluate humoral immunity post-vaccination is routinely practiced in immunocompromised patients. For instance, antibody testing for varicella, measles, mumps, rubella, and hepatitis B is commonly used to assess the need for booster vaccination. Serial antibody measurements have demonstrated waning antibody responses in kidney transplant recipients [18,35]. Therefore, antibody testing may have a role in guiding booster vaccination strategies. However, the optimal timing of antibody testing and whether antibody levels can be used to guide the timing of booster doses remain uncertain and should be evaluated prospectively. An antibody-guided vaccination strategy has previously been proposed in COVID-19-naïve kidney transplant recipients [17]. The cost-effectiveness of routine antibody testing to guide COVID-19 vaccination in kidney transplant recipients, compared with a strategy of fixed-interval vaccination, remains uncertain. Although the cost-effectiveness of the first booster vaccine has been estimated to range between $26,000-$116,000 per quality-adjusted life-year, subsequent boosters may not be cost-effective and could impose a financial burden, especially with emerging variants [45,46]. Thus, antibody-guided vaccination strategies warrant further economic evaluation to determine the most efficient approach to sustained protection in this high-risk population.

Our study has several strengths, including a relatively large sample size (n = 372). The study evaluated both clinical outcomes (subsequent COVID-19 infection and hospitalization) and quantitative measures of antibody response. However, several limitations should be acknowledged. First, the retrospective, single-center design may limit generalizability. In addition, antibody testing could not be performed in all eligible patients because of limited assay availability. Nevertheless, tested and untested eligible patients had similar baseline characteristics, including age, sex, race, and year of transplantation. The similarity of the measured baseline characteristics reduces concern regarding selection bias; however, residual selection bias related to unmeasured clinical characteristics or reasons for antibody testing cannot be excluded. Second, antibody titers were measured only once due to limited assay availability in Qatar, which precluded longitudinal assessment of antibody dynamics. We also cannot completely rule out the possibility that some patients had unrecognized or subclinical SARS-CoV-2 infection prior to antibody measurement. As a result, comparisons between seropositive and seronegative patients, or between those with low and high antibody levels, may have been affected, since some individuals categorized as seropositive or as having high antibody levels could have had antibody responses attributable to prior infection rather than vaccination. Third, the immunoassay used in this study (Elecsys®) has a measuring range of 0.40–250 U/mL, so antibody titers above 250 U/mL were reported as > 250 U/mL, limiting the ability to precisely differentiate between individuals with very high titers, including previously infected recipients with antibody levels above the assay’s upper measurement limit. Fourth, cellular immune responses (e.g., spike-specific CD4 + T cell interferon γ) were not assessed. Furthermore, variability in the timing of antibody measurement was inherent in the retrospective design, as many patients received their second vaccine dose before the anti–SARS-CoV-2 S immunoassay became available in Qatar. Finally, 96% of patients received the Pfizer-BioNTech vaccine, while only 4% received Moderna. Pfizer predominated because it was the first available vaccine in Qatar and kidney transplant recipients were prioritized for early vaccination. Although vaccine type was included in the regression analyses and was not significantly associated with the study outcomes, this imbalance may limit the generalizability of our findings to recipients of other mRNA vaccines.

In conclusion, understanding the immunologic response to the SARS-CoV-2 vaccine in kidney transplant recipients is important for improving protection against COVID-19 infection in this vulnerable population. Identifying transplant recipients with a low vaccine-induced antibody response may help guide the development of individualized immunization strategies. Further prospective studies are needed to determine whether antibody measurements could help guide individualized booster strategies in kidney transplant recipients.

## Supporting information

S1 Table Baseline characteristics of eligible kidney transplant recipients who were tested and not tested for anti-SARS-CoV-2 spike protein antibodies. (DOCX)
