# Supplementary material for: Assessment of coronavirus disease 2019 mRNA vaccine response in kidney transplant recipients
Source: PLoS One. 2026 Nov 4;21(11):e0359640. doi: 10.1371/journal.pone.0359640 (PMC13734576; doi:10.1371/journal.pone.0359640)
Supplement: Supplementary file 1 [file pone.0359640.s001.docx]

**S1 Table: Baseline characteristics of eligible kidney transplant recipients who were tested and not tested for anti-SARS-CoV-2 spike protein antibodies.**

| **Variable** | **Tested**  **(n=372)** | **Not Tested (n=205)** | **P value** |
| --- | --- | --- | --- |
| **Age, years, median (IQR)** | 52 (41-61) | 49 (40-61) | 0.32 |
| **Male gender, n (%)** | 268 (72) | 134 (65) | 0.09 |
| **Race, n (%)**  **Middle East**  **South Asia**  **East Asia**  **Africa**  **Others** | 251  58  24  34  5 | 127  34  14  23  7 | 0.40 |
| **Year of transplant, median (IQR)** | 2014 (2009-2016) | 2015 (2010-2017) | 0.08 |
