# Supplementary material for: Assessment of coronavirus disease 2019 mRNA vaccine response in kidney transplant recipients
Source: PLoS One. 2026 Nov 4;21(11):e0359640. doi: 10.1371/journal.pone.0359640 (PMC13734576; doi:10.1371/journal.pone.0359640)
Supplement: Supplementary file 2 [file pone.0359640.s003.docx]

**Response to Reviewers**

We sincerely thank the reviewers for their thorough and insightful comments. Their feedback has helped us clarify the rationale, strengthen the methodology, improve the interpretation of results, and enhance the overall presentation of our manuscript. We believe that addressing these comments has significantly improved the scientific rigor and clarity of the study, and we are grateful for their constructive guidance.

**Response to Reviewer 1:**

**1. Add more why it is important to do the said study in kidney transplant recipients. It is inadequately justified need for the study**

Thank you for this comment. We have added further justifications regarding the importance of our study in both “Introduction” and “Discussion” sections. We emphasized that kidney transplant recipients are uniquely susceptible to severe COVID-19 outcomes due to chronic immunosuppression, which leads to highly variable vaccine responses. Our study addresses this critical gap by identifying predictors of antibody development and magnitude of response. Furthermore, we highlight that simple seropositivity may not adequately reflect protection. Quantitative antibody titers are essential for accurate risk stratification, as patients with low titers are at higher risk for breakthrough infection and hospitalization.

**2. The use of 0.80 U/mL as the threshold for seroconversion and 121 U/mL as the cutoff between "low" and "high" titers seems arbitrary (based on median value). Authors should justify these thresholds and, if available, provide references or ROC analysis to support their clinical relevance.**

We appreciate this concern and have clarified the rationale in the “Methods” section of the revised manuscript. The 0.80 U/mL cutoff is the manufacturer-defined threshold for a positive result using the Elecsys® anti–SARS-CoV-2 S immunoassay. The 121 U/mL cutoff was used as an internal, data-driven stratification point corresponding to the median antibody titer among seropositive participants. This allowed us to test the hypothesis that antibody magnitude, not just seroconversion, is relevant to protection in kidney transplant recipients.

**3. Consider discussing how these cutoffs relate to protective immunity, especially since 250 U/mL is the upper assay limit.**

Thank you for your comment. We have clarified this in the Discussion*.* Our data showed that patients in the low-antibody titers had significantly higher infection and hospitalization rates compared to those with high-antibody titers, underscoring the potential link between quantitative response and protection. We also note that the limited measuring range of the Elecsys assay (0.40–250 U/mL) restricts our ability to differentiate very high antibody levels, which we have acknowledged as a study limitation.

**4. The analysis relies entirely on univariate comparisons. To strengthen the conclusions, particularly about predictors of poor vaccine response (e.g., age, sex, diabetes), a multivariate logistic regression should be included.**

We thank the reviewer for this valuable suggestion. A multivariable logistic regression analysis was performed and included in the revised manuscript. Two models were created: one identifying independent predictors of poor vaccine response (Table 3) and another identifying predictors of high- antibody titers (Table 5).

**5. Variables such as age, sex, diabetes, time from transplant, and immunosuppression regimen should be adjusted for in a single model.**

We agree and confirm that these variables were included in the multivariable model described above. The results and their implications are discussed in detail in the Discussion section.

**6. Antibody levels were measured between 14 days and a longer unspecified duration after the second vaccine dose. The time window may significantly affect titers.**

We thank the reviewer for this important observation. The variability in the timing of antibody measurement was an inherent limitation of our retrospective design, as most patients had received their second vaccine dose before the anti–SARS-CoV-2 S immunoassay became available in Qatar. To account for this, we included the variable “Days between second vaccine dose and antibody test” as a covariate in both univariable and multivariable analyses. Although this factor was not independently associated with antibody titers, its inclusion ensured appropriate adjustment for potential time-related effects.

**7. Consider adjusting for days post-vaccine to antibody test in the analysis or limiting to a tighter time window to reduce heterogeneity.**

We appreciate this valuable suggestion. While we acknowledge that a narrower time window would reduce heterogeneity, restricting our cohort in this way would have significantly reduced the statistical power of the study given its retrospective design. Instead, we analytically adjusted for time since vaccination,which we believe provides a reasonable balance.

**8. While the authors acknowledge the limitation, the discussion should emphasize the potential dissociation between humoral and cellular immunity, especially in transplant recipients. If any literature on CD4/CD8 response in similar populations is available, it should be briefly cited.**

We thank the reviewer for the excellent point regarding the potential dissociation between humoral and cellular immunity in kidney transplant recipients. We acknowledge that our study did not assess cellular immune responses, such as spike-specific CD4-T cell interferon-gamma. This limitation has been addressed in the revised Discussion section. We have also cited literature on CD4+ T-cell responses, highlighting differences in gender-related T-cell activity and the suppressive effects of anti-metabolite therapies (mycophenolate mofetil and azathioprine) on cellular immunity.

**9. The suggestion for routine post-vaccine antibody testing is reasonable but requires more nuanced discussion regarding feasibility, cost-effectiveness, and current guideline recommendations.**

We agree that a nuanced discussion is warranted. While a detailed cost-effectiveness analysis is beyond this study’s scope, we have strengthened the Discussion by referencing studies assessing the economic and practical aspects of booster vaccination and antibody testing. We emphasize that future research should assess the cost-effectiveness of incorporating post-vaccine antibody testing into routine care.

**10. Authors should also discuss the implications of their findings for booster vaccine strategies in transplant populations.**

We appreciate this suggestion and have expanded our Discussion accordingly. Our findings, which demonstrate lower infection rates among high-titer recipients, support a titer-guided booster approach in kidney transplant populations. We recommend considering antibody testing 4–6 months after vaccination, with an additional booster for patients who remain seronegative or have low titers. This approach directly addresses waning immunity and aims to maintain a titer level sufficient to confer protection against emerging SARS-CoV-2 variants.

**Minor Comments**

**1. The manuscript is generally well-written but contains minor grammatical and typographical issues (e.g., “the response to the COVID-19 mRNA vaccine… is essential to preventing…” should read “for preventing…”). A thorough proofreading is recommended.**

We thank the reviewer for this observation. The entire manuscript has been thoroughly proofread and edited for grammar, consistency, and clarity.

**2. Figures were not visible in the PDF; ensure they are uploaded correctly and readable.**

We apologize for the technical issue. All figures, including Figure 1 (Study Design) and Figure 2 (Time to COVID-19 Infection), have been correctly uploaded.

**3. Table 1 and Table 3 could benefit from improved formatting (e.g., consistent decimal places, avoid merged cells in PDF tables).**

We have reviewed all tables and standardized the formatting, including consistent decimal places and elimination of merged cells.

**4. References are appropriate and up-to-date. However, reference formatting should match the journal's guidelines (e.g., use of full journal names or abbreviations consistently).**

We have revised all references to conform fully to the journal’s formatting requirements.

**5. Data availability should be clarified. The manuscript states that data are within the manuscript and supplementary files, but none are provided in the PDF. Either include them or revise the statement.**

We thank the reviewer for this comment and have added the following clarification:

“The dataset used in this study is the property of the Qatar Ministry of Public Health (MOPH) and was provided to the researchers under a restricted-access agreement that prohibits sharing the dataset with third parties or making it publicly available. The data remain under restricted access to preserve patient confidentiality in compliance with national data privacy regulations. Researchers may request access to the dataset directly from the Ministry of Public Health via the Governmental Health Communication Center (https://www.moph.gov.qa/english/OurServices/eservices/Pages/Governmental-Health-Communication-Center.aspx). The raw data are therefore not publicly available, but aggregate and summary data are presented within the paper and its supplementary materials.”

**Response to Reviewer 2:**

**1. Why choose 121 U/mL as the high/low cutoff point? Is there any literature to support this classification?**

We thank the reviewer for this valuable comment. The 121 U/mL cutoff represented the median antibody titer among seropositive participants and was used as an internal, data-driven, exploratory stratification point to assess whether the magnitude of antibody response correlated with clinical outcomes such as COVID-19 infection or hospitalization. At the time of the study, no universally accepted or externally validated quantitative threshold existed to define protective immunity in kidney transplant recipients. Therefore, the median-based cutoff provided a pragmatic and unbiased means to explore these associations. This rationale has been clarified in the Methods section.

**2. In the study, 96% of participants received the Pfizer vaccine, while only 4% received the Moderna vaccine. Could this imbalance affect the generalizability of the results? Is it possible to increase the sample size of Moderna vaccine recipients to further validate the conclusions?**

Thank you for raising this point. Pfizer-BioNTech was the first available vaccine in Qatar, and kidney transplant recipients were prioritized for early vaccination among the general population. As a result, most of our patients received the Pfizer vaccine (96%). This study included all kidney transplant recipients who underwent antibody testing after receiving two vaccine doses; therefore, it was not possible to expand the Moderna subgroup. While vaccine type was included in both univariable and multivariable analyses and did not show statistically significant associations with outcomes, we have acknowledged this limitation in the Discussion section, noting that the small number of Moderna recipients may limit the generalizability of our findings to recipients of other mRNA vaccines.

**3. In this study, the authors only detected the antibody titers once. It would be better to provide long-term data to show the relationship between antibody titer change and COVID-19 risk.**

We thank the reviewer for this suggestion. We acknowledge that antibody titers were measured only once in our study, which limits the ability to assess longitudinal changes in antibody levels and their relationship with COVID-19 risk. This was due to the limited availability of the Elecsys® anti–SARS-CoV-2 S immunoassay in Qatar during the study period, which was prioritized for high-risk populations, including kidney transplant recipients. Despite this, our single post-vaccination measurement provides valuable insight into the association between antibody levels and COVID-19 incidence and severity in this population. We have now included this limitation in the Discussion section to clarify that long-term antibody kinetics could not be evaluated.

**4. The authors only evaluated the humoral responses, should cell-mediated responses be included in this study to get more fully assess of the vaccine, also can be compared whether gender is the only one significant factor associated with poor responses.**

We thank the reviewer for this important point. We agree that evaluating cell-mediated immunity, such as spike-specific CD4+ T-cell interferon-gamma responses, would provide a more comprehensive assessment of vaccine-induced immunity. However, due to the retrospective design of our study and the limitations of available clinical data, cellular immune responses were not assessed. We have now addressed this limitation in the revised Discussion. Additionally, we cite relevant literature on CD4+ T-cell responses in transplant recipients, highlighting gender-related differences in T-cell activity and the suppressive effects of anti-metabolite therapies, including mycophenolate mofetil and azathioprine, on both humoral and cellular immunity. This context supports our findings regarding gender and immunosuppressive regimen as significant factors associated with vaccine response.

**5. The authors conclude that gender is the only factor associated with poor response, it would be better to further investigate the potential reasons.**

We thank the reviewer for this observation. In the revised analysis, after performing multivariable regression, we identified additional factors associated with poor response to the COVID-19 mRNA vaccine beyond gender. Specifically, older age was also significantly associated with a higher risk of poor seroconversion. These findings are consistent with the concept of immunosenescence, where aging impairs both humoral and cellular immune responses. We have updated the Results and Discussion sections to reflect these additional factors and provide context regarding their biological plausibility.

**6. Most participants received a third dose of the vaccine. but the article did not discuss its impact on subsequent infection rates or antibody levels. Could the authors analyze and discuss its effect on antibody titers and infection rates?**

We thank the reviewer for this insightful comment. In our study, all antibody measurements were performed prior to administration of the third vaccine dose. Therefore, the impact of the third dose on subsequent antibody titers could not be directly evaluated. However, we did examine the uptake of the third dose across different patient groups (previous COVID-19 infection, seronegative, and seropositive), and found no significant differences between groups (P = 0.65).

**Minor concern:
1. Lines 113-115: “125 had low antibody (anti-SARS-CoV-2 S protein114 RBD antibody titer < 121 U/mL and 125 had high antibody titers (anti-SARS-CoV-2 S protein115 RBD antibody titer > 121 U/mL), as shown in Figure 1”. It should be “≥ 121 U/mL”, need to add “= 121” this situation.**

Thank you for your comment. This was corrected as suggested.

**2. Lines 152-154: The manuscript is inconsistent in its use of SARS-CoV-2, some lack the dash between SARS and CoV, it needs to be consistent throughout the manuscript**.

Thank you for your comment. This was corrected throughout the manuscript.
