# Supplementary material for: Assessment of coronavirus disease 2019 mRNA vaccine response in kidney transplant recipients
Source: PLoS One. 2026 Nov 4;21(11):e0359640. doi: 10.1371/journal.pone.0359640 (PMC13734576; doi:10.1371/journal.pone.0359640)
Supplement: Supplementary file 3 [file pone.0359640.s004.docx]

**Response to Reviewers**

We sincerely thank the reviewers and the editor for their careful evaluation of our manuscript and for their constructive comments. We are pleased that the reviewers consider the study technically sound and recognize the substantial improvements made in the previous revision.

We have carefully addressed all remaining comments, with particular attention to clarifying methodological details, refining reporting, and enhancing interpretation where requested. These revisions have further strengthened the clarity, transparency, and overall quality of the manuscript.

Below, we provide a detailed, point-by-point response to each comment and indicate the corresponding revisions made in the manuscript. All changes have been clearly marked in the revised version.

**Response to Academic Editor:**

**1) Clarifying the timing of prior SARS-CoV-2 infection relative to vaccination and antibody assessment (or acknowledging if unavailable).**

Thank you for your comment. This was clarified in the revised manuscript as follows (lines 179-191): Forty-five patients had a previous COVID-19 infection before anti‑SARS‑CoV‑2 S immunoassay testing. Of these, 21 patients had COVID-19 infection before vaccination, while 24 patients had infection after vaccination (6 patients developed infection after the first vaccine dose but before the second dose, and 18 patients developed infection after the second dose). The median time from infection to antibody testing was 381 days (IQR 289–421) for infections before vaccination, 112 days (IQR 99–131) for infections after the first dose, and 88.5 days (IQR 65.5–110.8) for infections after the second dose. The interval differed significantly across groups (p < 0.001). **Despite these differences in timing,** comparison of antibody levels across the three groups (**infection before vaccination, infection after the first dose but before the second dose, and infection after the second dose**) showed no statistically significant difference in antibody titers (median antibody **level** of 250 U/mL in all **groups, P = 0.54**). In addition, **Spearman correlation analysis** demonstrated **no significant association** between the time from infection to antibody testing and antibody titers (ρ = −0.064, P = 0.68).

**2) Clarifying the timing of third-dose vaccination relative to COVID-19 infection among those who developed infection.**

Thank you for your comment. This was clarified in the revised manuscript as follows (lines 222-244): During the follow-up period after anti‑SARS‑CoV‑2 S immunoassay testing, 74% of patients received a third COVID-19 mRNA vaccine (n=275), with no difference across groups (P = 0.65). Among patients with previous infection, 6 developed COVID-19 infection (3 after the third vaccine dose and 3 who did not receive the third dose). In seropositive patients, 66 patients developed infection (1 before the third dose, 38 after the third dose, and 27 who did not receive the third dose). In seronegative patients, 28 developed infection (22 after the third dose and 6 who did not receive the third dose). The time between the third vaccine dose and subsequent COVID-19 infection differed significantly between patients with previous infection and seronegative patients (median 118 vs 132 days, P = 0.04). Differences between previous infection and seropositive patients (118 versus 128 days, P = 0.14) and between seronegative and seropositive patients (128 versus 132 days, P = 0.06) were not statistically significant.

We also have added a row in Table 1 showing the distribution of the timing of the third vaccine dose among recipients who were infected with COVID-19.

**3) Revising Table 2 to report individual p-values for immunosuppressive medication categories**

Thank you for your comment. In the revised manuscript, we have included individual p-values for each immunosuppressive medication category in Table 2.

**4) Clarifying cohort selection and generalizability.**

Thank you for your comment. This was clarified in the revised manuscript as follows (lines 89-93): This was a retrospective cohort study of adult kidney transplant recipients who received two doses of COVID-19 mRNA vaccine after transplantation and had their anti‑SARS‑CoV‑2 S protein RBD antibodies measured at least 14 days after the second dose and before receiving a third dose. These criteria were established prior to testing and all patients meeting them who were tested before October 1, 2021, were included and followed until February 1, 2022.

Because all eligible patients were included consecutively, we believe the risk of selection bias was minimal in the study.

5) Revising use of the term “seroconversion” given the absence of baseline antibody testing.

Thank you for your comment. We agree that the absence of baseline antibody testing limits the ability to accurately define true seroconversion. Our goal was to identify patients with detectable antibodies after vaccination and those without detectable antibodies after vaccination. Therefore, we have revised the terminology throughout the manuscript and replaced “seroconversion” with “antibody status” or “seropositivity”.

**6) Discussing possible confounding by unrecognized/subclinical infection.**

Thank you for your comment. We have addressed this in the revised Discussion by adding the following statement (lines 42-427): We also cannot completely rule out the possibility that some patients had unrecognized or subclinical SARS-CoV-2 infection prior to antibody measurement, including infections with pre-Omicron variants. As a result, comparisons between seropositive versus seronegative patients, or between those with low versus high antibody levels, may be affected, since some individuals categorized as seropositive or having high antibody levels could have had antibody responses attributable to prior infection rather than vaccination.

**7) Justifying the use of logistic regression instead of time-to-event analysis (and providing a sensitivity analysis if feasible)**

Thank you for your comment. Logistic regression was used because the primary study outcomes—poor vaccine response (seronegativity) and high versus low antibody titers— were binary variables derived from a single antibody measurement obtained following the second vaccine dose, and the primary objective was to assess associations with antibody response. Although post-vaccination COVID-19 infections during follow-up were recorded, a time-to-event analysis using Cox proportional hazards regression was not performed, as this would have introduced several methodological complexities beyond the scope of the present study, including the need to incorporate the third vaccine dose as a time-varying exposure and to account for potential competing events—most notably death—neither of which were adequately captured in the available dataset. We have clarified this rationale in the Methods section of the revised manuscript (lines 161-169).

**8) Replacing “vaccine efficacy” with “vaccine effectiveness” where appropriate.**

Thank you for your comment. We have replaced “vaccine efficacy” with “vaccine effectiveness” throughout the manuscript to reflect the observational nature of our study.

**Response to Reviewer 2:**

**1) Does the timing of prior infection affect antibody titers? In the study, 45 patients with a history of previous infection had a median antibody titer of 250 U/mL (the upper limit of detection). Combining all past infections into a single group does not allow for definitive the time of infection (pre-vaccination? after first dose? after second dose?) But infections occurring before vaccination or after vaccination have entirely different biological significance for antibody maturation and memory response. It would be helpful if the authors could further clarify this aspect.**

Thank you for this important and insightful comment. We agree that the timing of prior infection in relation to vaccination may have different biological implications for antibody maturation and memory response. This was clarified in the revised manuscript as follows (lines 179-191): Forty-five patients had a previous COVID-19 infection before anti‑SARS‑CoV‑2 S immunoassay testing. Of these, 21 patients had COVID-19 infection before vaccination, while 24 patients had infection after vaccination (6 patients developed infection after the first vaccine dose but before the second dose, and 18 patients developed infection after the second dose). The median time from infection to antibody testing was 381 days (IQR 289–421) for infections before vaccination, 112 days (IQR 99–131) for infections after the first dose, and 88.5 days (IQR 65.5–110.8) for infections after the second dose. The interval differed significantly across groups (p < 0.001). **Despite these differences in timing,** comparison of antibody levels across the three groups (**infection before vaccination, infection after the first dose but before the second dose, and infection after the second dose**) showed no statistically significant difference in antibody titers (median antibody **level** of 250 U/mL in all **groups, P = 0.54**). In addition, **Spearman correlation analysis** demonstrated **no significant association** between the time from infection to antibody testing and antibody titers (ρ = −0.064, P = 0.68).

**Response to Reviewer 3:**

**1) The timing of administration of the third dose of vaccine in relation to COVID-19 infection. Taking a booster dose may reduce the risk of infection, and not all recipients who were eventually infected with COVID-19 had received a third dose prior to infection. Therefore, comparison of infection rate may be biased by the timing of third dose (i.e. before third dose, after third dose, or never had third dose). The authors could consider adding a few sentences or tables rows in the results section to show the distribution of timing of third dose among recipients who were infected with COVID-19.**

Thank you for your comment. This was clarified in the revised manuscript as follows (lines 222-244): During the follow-up period after anti‑SARS‑CoV‑2 S immunoassay testing, 74% of patients received a third COVID-19 mRNA vaccine (n=275), with no difference across groups (P = 0.65). Among patients with previous infection, 6 developed COVID-19 infection (3 after the third vaccine dose and 3 who did not receive the third dose). In seropositive patients, 66 patients developed infection (1 before the third dose, 38 after the third dose, and 27 who did not receive the third dose). In seronegative patients, 28 developed infection (22 after the third dose and 6 who did not receive the third dose). The time between the third vaccine dose and subsequent COVID-19 infection differed significantly between patients with previous infection and seronegative patients (median 118 vs 132 days, P = 0.04). Differences between previous infection and seropositive patients (118 versus 128 days, P = 0.14) and between seronegative and seropositive patients (128 versus 132 days, P = 0.06) were not statistically significant.

We also have added a row in Table 1 showing the distribution of the timing of the third vaccine dose among recipients who were infected with COVID-19.

**2) In Table 2, there should be 6 individual p-values reported for immunosuppression medications rather than a single p-value, as these categories are not mutually exclusive.**

Thank you for your comment. In the revised manuscript, we have included individual p-values for each immunosuppressive medication category in Table 2.

**Response to Reviewer 4:**

**1) The study included 372 kidney transplant recipients who received two doses of a COVID-19 mRNA vaccine and had anti–SARS-CoV-2 spike protein receptor-binding domain antibodies measured at least 14 days after the second dose and before receipt of a third dose. An important question is what proportion of all vaccinated kidney transplant recipients met this definition.** **If this represents a small subset, were there differences between recipients who qualified for inclusion and those who did not? This could affect the generalizability of the study’s findings to the broader kidney transplant population.**

Thank you for your comment. This was clarified in the revised manuscript as follows (lines 89-93): This was a retrospective cohort study of adult kidney transplant recipients who received two doses of COVID-19 mRNA vaccine after transplantation and had their anti‑SARS‑CoV‑2 S protein RBD antibodies measured at least 14 days after the second dose and before receiving a third dose. These criteria were established prior to testing and all patients meeting them who were tested before October 1, 2021, were included and followed until February 1, 2022.

Because all eligible patients were included consecutively, we believe the risk of selection bias was minimal in the study.

**2) In Line 145, the authors state: “Of the remaining 327 patients, 250 (76%) had seroconversion.” Because baseline antibody levels were not measured, how can the authors be certain that positive antibody results represent true seroconversion? Is it possible that some patients had detectable antibodies prior to vaccination due to previously unrecognized SARS-CoV-2 infection?**

Thank you for your comment. We agree that the absence of baseline antibody testing limits the ability to accurately define true seroconversion. Our goal was to identify patients with detectable antibodies after vaccination and those without detectable antibodies after vaccination. Therefore, we have revised the terminology throughout the manuscript and replaced “seroconversion” with “antibody status” or “seropositivity”.

**3) Relatedly, can the authors comment on the possibility of subclinical COVID-19 infection prior to the emergence of the Omicron variant? Pre-Omicron variants would have been circulating during the interval between the second vaccine dose and antibody assessment. The comparisons between seropositive versus seronegative groups, as well as low versus high antibody-level groups, would be more robust if subclinical infection were unlikely during this period. Otherwise, some individuals categorized as seropositive or having high antibody levels may have had antibody responses attributable to prior infection rather than vaccination, which could confound the observed protective associations.**

Thank you for your comment. We have addressed this in the revised Discussion by adding the following statement (lines 422-427): We also cannot completely rule out the possibility that some patients had unrecognized or subclinical SARS-CoV-2 infection prior to antibody measurement, including infections with pre-Omicron variants. As a result, comparisons between seropositive versus seronegative patients, or between those with low versus high antibody levels, may be affected, since some individuals categorized as seropositive or having high antibody levels could have had antibody responses attributable to prior infection rather than vaccination.

**4) It appears that time-to-COVID-19 event data were available. If so, Cox proportional hazards regression might be a more appropriate analytical approach than logistic regression. Is there a reason logistic regression was chosen instead of a time-to-event analysis?**

Thank you for your comment. Logistic regression was used because the primary study outcomes—poor vaccine response (seronegativity) and high versus low antibody titers— were binary variables derived from a single antibody measurement obtained following the second vaccine dose, and the primary objective was to assess associations with antibody response. Although post-vaccination COVID-19 infections during follow-up were recorded, a time-to-event analysis using Cox proportional hazards regression was not performed, as this would have introduced several methodological complexities beyond the scope of the present study, including the need to incorporate the third vaccine dose as a time-varying exposure and to account for potential competing events—most notably death—neither of which were adequately captured in the available dataset. We have clarified this rationale in the Methods section of the revised manuscript (lines 161-169).

**5) In Line 330, the term “vaccine effectiveness” should be used instead of “vaccine efficacy,” as the latter is more appropriately reserved for randomized controlled trials.**

Thank you for your comment. We have replaced “vaccine efficacy” with “vaccine effectiveness” throughout the manuscript to reflect the observational nature of our study.
