# Supplementary material for: Assessment of coronavirus disease 2019 mRNA vaccine response in kidney transplant recipients
Source: PLoS One. 2026 Nov 4;21(11):e0359640. doi: 10.1371/journal.pone.0359640 (PMC13734576; doi:10.1371/journal.pone.0359640)
Supplement: Supplementary file 4 [file pone.0359640.s005.docx]

**Response to Reviewers**

**Response to Reviewer 4:**

This study included kidney transplant recipients who had spike protein antibody levels checked before their third COVID-19 vaccine dose. It was not standard practice to check spike protein antibodies before a third COVID-19 mRNA vaccine was given. Since this was a retrospective study, one could not go back in time and check anti-SARS-CoV-2 spike protein antibodies in patients in whom these antibodies were not checked. Unless there was a national or an institutional policy not to administer a third dose of vaccine until spike antibody levels were checked, there would surely have been patients who did not have spike antibody levels tested before their third vaccine dose. So the question is what proportion of kidney transplant recipients had spike protein antibody levels checked between their second and third dose? Were these people systematically different from those who did not get such testing done? Was there something about these people that prompted spike antibody level testing before their third vaccine dose? If so, there may be questions about generalizability.
The fact that all "eligible" kidney transplant patients were included consecutively does not necessarily mean the study can be generalized to the broader kidney transplant population, which would include "ineligible" kidney transplant recipients, that is those who did not have spike antibody testing prior to receiving a third dose of vaccine.

We sincerely thank the reviewer for this important and insightful comment regarding the potential for selection bias and generalizability. As correctly noted, it was not standard clinical practice to measure spike protein antibody levels prior to administration of a third COVID-19 mRNA vaccine dose, and there was no national or institutional policy requiring antibody testing before booster vaccination. We also acknowledge that inclusion of "eligible" patients does not automatically ensure generalizability to the broader kidney transplant population, which includes patients who did not undergo antibody testing prior to receiving a third dose.

Regarding the proportion of kidney transplant recipients who underwent spike protein antibody testing performed between the second and third vaccine doses, the anti-SARS-CoV-2 S protein RBD immunoassay was available in Qatar in limited quantities between June and September 2021. In addition, the national COVID-19 booster vaccination program was initiated on 24 August 2021.

Overall, 372 of 577 eligible adult kidney transplant recipients (64%) underwent anti-SARS‑CoV‑2 S protein RBD antibody testing after receiving two doses of the COVID-19 mRNA vaccine and prior to receipt of the third dose. Baseline characteristics were comparable between tested and untested eligible patients, with no statistically significant differences in age, gender, race, or year of transplantation, suggesting that selection bias is unlikely to have significantly affected the findings (Supplementary Table 1).

This point has been clarified in the revised manuscript, including the Methods, Results, and Discussion (limitations) sections (Lines 87-93; 152-158; 374-378) and Figure 1.
