# Supplementary material for: Assessment of coronavirus disease 2019 mRNA vaccine response in kidney transplant recipients
Source: PLoS One. 2026 Nov 4;21(11):e0359640. doi: 10.1371/journal.pone.0359640 (PMC13734576; doi:10.1371/journal.pone.0359640)
Supplement: Supplementary file 5 [file pone.0359640.s006.docx]

**Response to the Academic Editor**

Dear Dr. Berber,
Thank you for your careful review of our manuscript and for the opportunity to submit a revised version. We appreciate your detailed comments and recommendations, which helped us improve the statistical reporting, clarify the interpretation of our findings, and improve the overall consistency of the manuscript.

We have carefully addressed each of the editorial comments and revised the manuscript accordingly. Below, we provide a detailed, point-by-point response to each comment and indicate the corresponding revisions made in the manuscript. All changes have been clearly marked in the revised version.

**Response to Additional Editor Comments:**

**1) The Methods state that Cox regression was not performed, but Section 3.5 and Figure 2 nevertheless report “time to infection” comparisons and a P value of 0.007. The statistical procedure is not identified. The authors must state: Whether Kaplan–Meier analysis and a log-rank test were used. How participants without infection were censored. The censoring date and whether death, loss to follow-up, or third-dose vaccination affected censoring. Whether the reported P value is an overall test or a pairwise comparison. Whether pairwise comparisons were adjusted for multiple testing. These details are required for Section 3.5 and Figure 2.**

Thank you for your valuable comment. We have revised Section 2.4 (Statistical analysis) of the Materials and Methods and Section 3.5 (Time to COVID-19 infection) of the Results to explicitly describe the time-to-infection analysis. Time to COVID-19 infection was analyzed using Kaplan–Meier survival analysis, with the date of the anti-SARS-CoV-2 S immunoassay test as time zero and subsequent COVID-19 infection as the event. Participants who did not develop COVID-19 infection were censored on February 1, 2022. No participants were censored because of death, loss to follow-up, or receipt of a third vaccine dose. The Kaplan–Meier curves for the four groups were compared using an overall log-rank test. After verification and reanalysis of the data, the overall log-rank test yielded **P = 0.021**; accordingly, the previously reported P value of 0.007 has been corrected. No pairwise log-rank comparisons were performed; therefore, no adjustment for multiple comparisons was required. Figure 2, including the figure legend, has been revised accordingly.

**2) The manuscript states that antibody levels were evaluated for their ability to “predict” infection and severity. However, the comparisons of subsequent infection are primarily unadjusted proportions. Moreover, 74% of participants received a third vaccine dose during follow-up, and the third dose was not incorporated as a time-varying exposure. Consequently, the study demonstrates an association, not independent prediction, vaccine effectiveness, or causation. The authors should: Replace “predicting the incidence and severity” with “examining associations with subsequent infection and hospitalization.” Avoid stating that binding antibodies “remained effective against Omicron.” Avoid referring to the measured antibodies as neutralizing antibodies because neutralization was not measured. Remove or substantially soften the recommendation to test antibodies at 4–6 months and administer boosters according to the result. This should be presented only as a hypothesis requiring prospective evaluation. Replace “hospitalization for severe disease” unless a validated definition of severe COVID-19 was applied.**

Thank you for your valuable comment. We have revised the manuscript throughout to ensure that the findings are presented as associations rather than as independent prediction, vaccine effectiveness, or causation. We replaced “predicting the incidence and severity” with “examining associations with subsequent infection and hospitalization,” and references to prediction were changed to association where appropriate. We also removed the statement that binding antibodies “remained effective against Omicron” and removed references to the measured antibodies as neutralizing antibodies, as neutralization was not assessed in this study. The term “hospitalization for severe disease” was replaced with “hospitalization” where appropriate.

The previous recommendation to perform antibody testing at 4–6 months and administer booster doses according to antibody levels was removed. The Discussion was revised to present antibody-guided booster vaccination as a potential strategy requiring prospective evaluation. We now state that antibody testing may have a role in guiding booster vaccination strategies; however, the optimal timing of antibody testing and whether antibody levels can be used to guide the timing of booster doses remain uncertain and should be evaluated prospectively. The Conclusion was also revised accordingly. We now state that identifying transplant recipients with a low vaccine-induced antibody response may help guide the development of individualized immunization strategies, while emphasizing that further prospective studies are needed to determine whether antibody measurements could help guide individualized booster strategies and optimize protection in kidney transplant recipients.

**3) The response to Reviewer 4 is substantially satisfactory, but similarity in age, sex, geographic/racial category, and transplant year does not eliminate selection bias. Clinical factors such as immunosuppressive regimen, renal function, comorbidities, healthcare utilization, or clinician reasons for ordering testing may still differ. The limitation should read: “The similarity of the measured baseline characteristics reduces concern regarding selection bias; however, residual selection bias related to unmeasured clinical characteristics or reasons for antibody testing cannot be excluded.”**

Thank you for your valuable comment. We agree that similarity in the measured baseline characteristics does not eliminate the possibility of selection bias related to unmeasured factors. We have revised the Limitations section accordingly. The manuscript now states: “The similarity of the measured baseline characteristics reduces concern regarding selection bias; however, residual selection bias related to unmeasured clinical characteristics or reasons for antibody testing cannot be excluded.”

**4) Define how COVID-19 infection was diagnosed—PCR, antigen testing, clinical diagnosis, or another criterion.**

Thank you for your valuable comment. We have clarified the criteria used to diagnose COVID-19 infection in Section 2.3 (Data collection) of the Materials and Methods. All COVID-19 infections included in the study were PCR-confirmed. During the pre-Omicron period, diagnosis was based exclusively on PCR testing. During the Omicron-predominant period, rapid antigen testing was also used, but positive antigen test results required confirmation by PCR.

**5) Explain how infections were attributed to Omicron, or revise the text to “infections occurring during the Omicron-predominant period.”**

Thank you for your valuable comment. We agree that the infections cannot be definitively attributed to the Omicron variant. Therefore, we have revised the manuscript as suggested and replaced references to Omicron infections with “infections occurring during the Omicron-predominant period” where appropriate.

**6) Include the informed-consent waiver in the submission-system ethics statement, consistent with the Methods.**

Thank you for your valuable comment. We have updated the submission-system ethics statement to include the informed-consent waiver, consistent with the Methods section.

**7) Correct language errors including “antibodyd,” “anit-SARS-CoV-2,” “A Poor response,” and inconsistent capitalization.**

Thank you for your valuable comment. The identified language errors and inconsistencies in capitalization have been corrected in the revised manuscript.

**8) Several errors need direct verification against the source dataset:**

**Lines 213–214: The manuscript says female sex was more common in the seronegative group “75% versus 62%.” Those are the reported male percentages and are also presented in the reverse order. Based on Table 2, female percentages appear to be 38% versus 25%.**

Thank you for your valuable comment. The data were verified, and we confirmed that the previously reported percentages represented male rather than female participants. The percentages have been corrected in the revised manuscript to 38% versus 25%.

**Lines 240–242:** **Diabetes is described as “69% versus 53%,” but Table 4 reports** **69 patients (55%) versus 53 patients (42%)**.

Thank you for your valuable comment. The data were verified, and the percentages have been corrected in the revised manuscript to 55% versus 42%.

**Lines 243–244:** **The text gives P = 0.02 for the interval between dose 2 and antibody testing, while Table 4 gives** **P = 0.002**.

Thank you for your valuable comment. The data were verified, and the statistical analysis was repeated, confirming a P value of 0.002. This has been corrected in the revised manuscript.

**Table 4: “Months from transplant” is reported as 86.8 versus 86.9 months with P = 0.02, while Table 5 reports no association after median categorization. The P value should be checked and the significant result discussed if correct.**

Thank you for your valuable comment. We reviewed the statistical analysis and identified that an unpaired t-test had initially been used in Table 4 to compare months from transplant. Upon reviewing the distribution of the data, we found that the distribution of months from transplant was highly skewed, making an unpaired t-test inappropriate. We repeated the analysis using the Mann–Whitney U test, which is more appropriate for this non-normally distributed variable. The analysis yielded a P value of 0.51, and the P value in Table 4 has consequently been corrected from 0.02 to 0.51 in the revised manuscript.

**Table 1 footnote:** **It incorrectly states that one-way ANOVA was used for categorical variables. Categorical comparisons should be identified as chi-square or Fisher’s exact tests. The authors should identify precisely whether ANOVA or Kruskal–Wallis testing was used for each continuous variable.**

Thank you for your valuable comment. We have corrected the statistical methods reported in the Table 1 footnote. Categorical variables were compared using the chi-square test or Fisher’s exact test, as appropriate. For continuous variables, one-way ANOVA was used for variables with an approximately normal distribution, whereas the Kruskal–Wallis test was used for variables with a non-normal or markedly skewed distribution. Specifically, one-way ANOVA was used for age, while the Kruskal–Wallis test was used for SARS-CoV-2 antibody titer, days between the second vaccine dose and immunoassay, days between immunoassay and COVID-19 infection, and days between the third vaccine dose and COVID-19 infection. The Table 1 footnote has been revised, and the statistical methods have also been corrected accordingly in Section 2.4 (Statistical analysis) of the Materials and Methods.
