# Supplementary material for: Assessment of coronavirus disease 2019 mRNA vaccine response in kidney transplant recipients
Source: PLoS One. 2026 Nov 4;21(11):e0359640. doi: 10.1371/journal.pone.0359640 (PMC13734576; doi:10.1371/journal.pone.0359640)
Supplement: Peer review history

# Peer review: Decision Letter 0

**DOI:** 10.1371/journal.pone.0359640.r001

**Authors:** Caijun Sun

20 Aug 2025

Dear Dr. Alkadi,

Please submit your revised manuscript by Oct 04 2025 11:59PM. If you will need more time than this to complete your revisions, please reply to this message or contact the journal office at plosone@plos.org. When you're ready to submit your revision, log on to https://www.editorialmanager.com/pone/ and select the 'Submissions Needing Revision' folder to locate your manuscript file.

- A rebuttal letter that responds to each point raised by the academic editor and reviewer(s). You should upload this letter as a separate file labeled 'Response to Reviewers'.
- A marked-up copy of your manuscript that highlights changes made to the original version. You should upload this as a separate file labeled 'Revised Manuscript with Track Changes'.
- An unmarked version of your revised paper without tracked changes. You should upload this as a separate file labeled 'Manuscript'.

If you would like to make changes to your financial disclosure, please include your updated statement in your cover letter. Guidelines for resubmitting your figure files are available below the reviewer comments at the end of this letter.

If applicable, we recommend that you deposit your laboratory protocols in protocols.io to enhance the reproducibility of your results. Protocols.io assigns your protocol its own identifier (DOI) so that it can be cited independently in the future. For instructions see: https://journals.plos.org/plosone/s/submission-guidelines#loc-laboratory-protocols. Additionally, PLOS ONE offers an option for publishing peer-reviewed Lab Protocol articles, which describe protocols hosted on protocols.io. Read more information on sharing protocols at https://plos.org/protocols?utm_medium=editorial-email&utm_source=authorletters&utm_campaign=protocols.

We look forward to receiving your revised manuscript.

Kind regards,

Caijun Sun

Academic Editor

PLOS ONE

Journal Requirements:

When submitting your revision, we need you to address these additional requirements.

1. Please ensure that your manuscript meets PLOS ONE's style requirements, including those for file naming. The PLOS ONE style templates can be found at https://journals.plos.org/plosone/s/file?id=wjVg/PLOSOne_formatting_sample_main_body.pdf and https://journals.plos.org/plosone/s/file?id=ba62/PLOSOne_formatting_sample_title_authors_affiliations.pdf

2. We note that you have indicated that there are restrictions to data sharing for this study. For studies involving human research participant data or other sensitive data, we encourage authors to share de-identified or anonymized data. However, when data cannot be publicly shared for ethical reasons, we allow authors to make their data sets available upon request. For information on unacceptable data access restrictions, please see http://journals.plos.org/plosone/s/data-availability#loc-unacceptable-data-access-restrictions.

Before we proceed with your manuscript, please address the following prompts:

a) If there are ethical or legal restrictions on sharing a de-identified data set, please explain them in detail (e.g., data contain potentially identifying or sensitive patient information, data are owned by a third-party organization, etc.) and who has imposed them (e.g., a Research Ethics Committee or Institutional Review Board, etc.). Please also provide contact information for a data access committee, ethics committee, or other institutional body to which data requests may be sent.

b) If there are no restrictions, please upload the minimal anonymized data set necessary to replicate your study findings to a stable, public repository and provide us with the relevant URLs, DOIs, or accession numbers. Please see http://www.bmj.com/content/340/bmj.c181.long for guidelines on how to de-identify and prepare clinical data for publication. For a list of recommended repositories, please see https://journals.plos.org/plosone/s/recommended-repositories. You also have the option of uploading the data as Supporting Information files, but we would recommend depositing data directly to a data repository if possible.

Please update your Data Availability statement in the submission form accordingly.

3. We note that your Data Availability Statement is currently as follows: All relevant data are within the manuscript and its Supporting Information files.

Please confirm at this time whether or not your submission contains all raw data required to replicate the results of your study. Authors must share the “minimal data set” for their submission. PLOS defines the minimal data set to consist of the data required to replicate all study findings reported in the article, as well as related metadata and methods (https://journals.plos.org/plosone/s/data-availability#loc-minimal-data-set-definition).

For example, authors should submit the following data:

- The values behind the means, standard deviations and other measures reported;

- The values used to build graphs;

- The points extracted from images for analysis.

Authors do not need to submit their entire data set if only a portion of the data was used in the reported study.

If your submission does not contain these data, please either upload them as Supporting Information files or deposit them to a stable, public repository and provide us with the relevant URLs, DOIs, or accession numbers. For a list of recommended repositories, please see https://journals.plos.org/plosone/s/recommended-repositories.

If there are ethical or legal restrictions on sharing a de-identified data set, please explain them in detail (e.g., data contain potentially sensitive information, data are owned by a third-party organization, etc.) and who has imposed them (e.g., an ethics committee). Please also provide contact information for a data access committee, ethics committee, or other institutional body to which data requests may be sent. If data are owned by a third party, please indicate how others may request data access.

4. When completing the data availability statement of the submission form, you indicated that you will make your data available on acceptance. We strongly recommend all authors decide on a data sharing plan before acceptance, as the process can be lengthy and hold up publication timelines. Please note that, though access restrictions are acceptable now, your entire data will need to be made freely accessible if your manuscript is accepted for publication. This policy applies to all data except where public deposition would breach compliance with the protocol approved by your research ethics board. If you are unable to adhere to our open data policy, please kindly revise your statement to explain your reasoning and we will seek the editor's input on an exemption. Please be assured that, once you have provided your new statement, the assessment of your exemption will not hold up the peer review process.

5. If the reviewer comments include a recommendation to cite specific previously published works, please review and evaluate these publications to determine whether they are relevant and should be cited. There is no requirement to cite these works unless the editor has indicated otherwise.

[Note: HTML markup is below. Please do not edit.]

Reviewers' comments:

Reviewer's Responses to Questions

**Comments to the Author**

1. Is the manuscript technically sound, and do the data support the conclusions?

Reviewer #1: Yes

Reviewer #2: Yes

**********

2. Has the statistical analysis been performed appropriately and rigorously?

Reviewer #1: Yes

Reviewer #2: Yes

**********

3. Have the authors made all data underlying the findings in their manuscript fully available?

The PLOS Data policy

Reviewer #1: No

Reviewer #2: Yes

**********

4. Is the manuscript presented in an intelligible fashion and written in standard English?

Reviewer #1: Yes

Reviewer #2: Yes

**********

Reviewer #1: Major Comments

1. Add more why it is important to do the said study in kidney transplant recipients. It is inadequately justified need for the study

2. The use of 0.80 U/mL as the threshold for seroconversion and 121 U/mL as the cutoff between "low" and "high" titers seems arbitrary (based on median value). Authors should justify these thresholds and, if available, provide references or ROC analysis to support their clinical relevance.

3. Consider discussing how these cutoffs relate to protective immunity, especially since 250 U/mL is the upper assay limit.

4. The analysis relies entirely on univariate comparisons. To strengthen the conclusions, particularly about predictors of poor vaccine response (e.g., age, sex, diabetes), a multivariate logistic regression should be included.

5. Variables such as age, sex, diabetes, time from transplant, and immunosuppression regimen should be adjusted for in a single model.

6. Antibody levels were measured between 14 days and a longer unspecified duration after the second vaccine dose. The time window may significantly affect titers.

7. Consider adjusting for days post-vaccine to antibody test in the analysis or limiting to a tighter time window to reduce heterogeneity.

8. While the authors acknowledge the limitation, the discussion should emphasize the potential dissociation between humoral and cellular immunity, especially in transplant recipients. If any literature on CD4/CD8 response in similar populations is available, it should be briefly cited.

9. The suggestion for routine post-vaccine antibody testing is reasonable but requires more nuanced discussion regarding feasibility, cost-effectiveness, and current guideline recommendations.

10. Authors should also discuss the implications of their findings for booster vaccine strategies in transplant populations.

Minor Comments

1. The manuscript is generally well-written but contains minor grammatical and typographical issues (e.g., “the response to the COVID-19 mRNA vaccine… is essential to preventing…” should read “for preventing…”). A thorough proofreading is recommended.

2. Figures were not visible in the PDF; ensure they are uploaded correctly and readable.

3. Table 1 and Table 3 could benefit from improved formatting (e.g., consistent decimal places, avoid merged cells in PDF tables).

4. References are appropriate and up-to-date. However, reference formatting should match the journal's guidelines (e.g., use of full journal names or abbreviations consistently).

5. Data availability should be clarified. The manuscript states that data are within the manuscript and supplementary files, but none are provided in the PDF. Either include them or revise the statement.

Recommendation

Major revision

The study is important and potentially publishable, but the lack of multivariate analysis, unclear antibody thresholds, and limited immunological context weaken the current version. Addressing these concerns would significantly improve the manuscript's scientific rigor and impact.

Reviewer #2: This retrospective analysis of 372 kidney transplant recipients assessed their antibody response to the COVID-19 mRNA vaccine, explored factors influencing immunogenicity, and investigated the predictive role of antibody levels on the incidence and severity of COVID-19 infection.

1. Why choose 121 U/mL as the high/low cutoff point? Is there any literature to support this classification?

2. In the study, 96% of participants received the Pfizer vaccine, while only 4% received the Moderna vaccine. Could this imbalance affect the generalizability of the results? Is it possible to increase the sample size of Moderna vaccine recipients to further validate the conclusions?

3. In this study, the authors only detected the antibody titers once. It would be better to provide long-term data to show the relationship between antibody titer change and COVID-19 risk.

4. The authors only evaluated the humoral responses, should cell-mediated responses be included in this study to get more fully assess of the vaccine, also can be compared whether gender is the only one significant factor associated with poor responses.

5. The authors conclude that gender is the only factor associated with poor response, it would be better to further investigate the potential reasons.

6. Most participants received a third dose of the vaccine. but the article did not discuss its impact on subsequent infection rates or antibody levels. Could the authors analyze and discuss its effect on antibody titers and infection rates?

Minor concern:

1. Lines 113-115: “125 had low antibody (anti-SARS-CoV-2 S protein114 RBD antibody titer < 121 U/mL and 125 had high antibody titers (anti-SARS-CoV-2 S protein115 RBD antibody titer > 121 U/mL), as shown in Figure 1”. It should be “≥ 121 U/mL”, need to add “= 121” this situation.

2. Lines 152-154: The manuscript is inconsistent in its use of SARS-CoV-2, some lack the dash between SARS and CoV, it needs to be consistent throughout the manuscript.

**********

what does this mean?). If published, this will include your full peer review and any attached files.

If you choose “no”, your identity will remain anonymous but your review may still be made public.

**Do you want your identity to be public for this peer review?**  For information about this choice, including consent withdrawal, please see our Privacy Policy

Reviewer #1: **Yes:**  Dr Dhiraj Agarwal

Reviewer #2: No

**********

[NOTE: If reviewer comments were submitted as an attachment file, they will be attached to this email and accessible via the submission site. Please log into your account, locate the manuscript record, and check for the action link "View Attachments". If this link does not appear, there are no attachment files.]

While revising your submission, please upload your figure files to the Preflight Analysis and Conversion Engine (PACE) digital diagnostic tool, https://pacev2.apexcovantage.com/. PACE helps ensure that figures meet PLOS requirements. To use PACE, you must first register as a user. Registration is free. Then, login and navigate to the UPLOAD tab, where you will find detailed instructions on how to use the tool. If you encounter any issues or have any questions when using PACE, please email PLOS at figures@plos.org. Please note that Supporting Information files do not need this step.

# Author comment: Author response to Decision Letter 1

**DOI:** 10.1371/journal.pone.0359640.r002

7 Nov 2025

Response to Reviewers

We sincerely thank the reviewers for their thorough and insightful comments. Their feedback has helped us clarify the rationale, strengthen the methodology, improve the interpretation of results, and enhance the overall presentation of our manuscript. We believe that addressing these comments has significantly improved the scientific rigor and clarity of the study, and we are grateful for their constructive guidance.

Response to Reviewer 1:

1. Add more why it is important to do the said study in kidney transplant recipients. It is inadequately justified need for the study

Thank you for this comment. We have added further justifications regarding the importance of our study in both “Introduction” and “Discussion” sections. We emphasized that kidney transplant recipients are uniquely susceptible to severe COVID-19 outcomes due to chronic immunosuppression, which leads to highly variable vaccine responses. Our study addresses this critical gap by identifying predictors of antibody development and magnitude of response. Furthermore, we highlight that simple seropositivity may not adequately reflect protection. Quantitative antibody titers are essential for accurate risk stratification, as patients with low titers are at higher risk for breakthrough infection and hospitalization.

2. The use of 0.80 U/mL as the threshold for seroconversion and 121 U/mL as the cutoff between "low" and "high" titers seems arbitrary (based on median value). Authors should justify these thresholds and, if available, provide references or ROC analysis to support their clinical relevance.

We appreciate this concern and have clarified the rationale in the “Methods” section of the revised manuscript. The 0.80 U/mL cutoff is the manufacturer-defined threshold for a positive result using the Elecsys® anti–SARS-CoV-2 S immunoassay. The 121 U/mL cutoff was used as an internal, data-driven stratification point corresponding to the median antibody titer among seropositive participants. This allowed us to test the hypothesis that antibody magnitude, not just seroconversion, is relevant to protection in kidney transplant recipients.

3. Consider discussing how these cutoffs relate to protective immunity, especially since 250 U/mL is the upper assay limit.

Thank you for your comment. We have clarified this in the Discussion. Our data showed that patients in the low-antibody titers had significantly higher infection and hospitalization rates compared to those with high-antibody titers, underscoring the potential link between quantitative response and protection. We also note that the limited measuring range of the Elecsys assay (0.40–250 U/mL) restricts our ability to differentiate very high antibody levels, which we have acknowledged as a study limitation.

4. The analysis relies entirely on univariate comparisons. To strengthen the conclusions, particularly about predictors of poor vaccine response (e.g., age, sex, diabetes), a multivariate logistic regression should be included.

We thank the reviewer for this valuable suggestion. A multivariable logistic regression analysis was performed and included in the revised manuscript. Two models were created: one identifying independent predictors of poor vaccine response (Table 3) and another identifying predictors of high- antibody titers (Table 5).

5. Variables such as age, sex, diabetes, time from transplant, and immunosuppression regimen should be adjusted for in a single model.

We agree and confirm that these variables were included in the multivariable model described above. The results and their implications are discussed in detail in the Discussion section.

6. Antibody levels were measured between 14 days and a longer unspecified duration after the second vaccine dose. The time window may significantly affect titers.

We thank the reviewer for this important observation. The variability in the timing of antibody measurement was an inherent limitation of our retrospective design, as most patients had received their second vaccine dose before the anti–SARS-CoV-2 S immunoassay became available in Qatar. To account for this, we included the variable “Days between second vaccine dose and antibody test” as a covariate in both univariable and multivariable analyses. Although this factor was not independently associated with antibody titers, its inclusion ensured appropriate adjustment for potential time-related effects.

7. Consider adjusting for days post-vaccine to antibody test in the analysis or limiting to a tighter time window to reduce heterogeneity.

We appreciate this valuable suggestion. While we acknowledge that a narrower time window would reduce heterogeneity, restricting our cohort in this way would have significantly reduced the statistical power of the study given its retrospective design. Instead, we analytically adjusted for time since vaccination,which we believe provides a reasonable balance.

8. While the authors acknowledge the limitation, the discussion should emphasize the potential dissociation between humoral and cellular immunity, especially in transplant recipients. If any literature on CD4/CD8 response in similar populations is available, it should be briefly cited.

We thank the reviewer for the excellent point regarding the potential dissociation between humoral and cellular immunity in kidney transplant recipients. We acknowledge that our study did not assess cellular immune responses, such as spike-specific CD4-T cell interferon-gamma. This limitation has been addressed in the revised Discussion section. We have also cited literature on CD4+ T-cell responses, highlighting differences in gender-related T-cell activity and the suppressive effects of anti-metabolite therapies (mycophenolate mofetil and azathioprine) on cellular immunity.

9. The suggestion for routine post-vaccine antibody testing is reasonable but requires more nuanced discussion regarding feasibility, cost-effectiveness, and current guideline recommendations.

We agree that a nuanced discussion is warranted. While a detailed cost-effectiveness analysis is beyond this study’s scope, we have strengthened the Discussion by referencing studies assessing the economic and practical aspects of booster vaccination and antibody testing. We emphasize that future research should assess the cost-effectiveness of incorporating post-vaccine antibody testing into routine care.

10. Authors should also discuss the implications of their findings for booster vaccine strategies in transplant populations.

We appreciate this suggestion and have expanded our Discussion accordingly. Our findings, which demonstrate lower infection rates among high-titer recipients, support a titer-guided booster approach in kidney transplant populations. We recommend considering antibody testing 4–6 months after vaccination, with an additional booster for patients who remain seronegative or have low titers. This approach directly addresses waning immunity and aims to maintain a titer level sufficient to confer protection against emerging SARS-CoV-2 variants.

Minor Comments

1. The manuscript is generally well-written but contains minor grammatical and typographical issues (e.g., “the response to the COVID-19 mRNA vaccine… is essential to preventing…” should read “for preventing…”). A thorough proofreading is recommended.

We thank the reviewer for this observation. The entire manuscript has been thoroughly proofread and edited for grammar, consistency, and clarity.

2. Figures were not visible in the PDF; ensure they are uploaded correctly and readable.

We apologize for the technical issue. All figures, including Figure 1 (Study Design) and Figure 2 (Time to COVID-19 Infection), have been correctly uploaded.

3. Table 1 and Table 3 could benefit from improved formatting (e.g., consistent decimal places, avoid merged cells in PDF tables).

We have reviewed all tables and standardized the formatting, including consistent decimal places and elimination of merged cells.

4. References are appropriate and up-to-date. However, reference formatting should match the journal's guidelines (e.g., use of full journal names or abbreviations consistently).

We have revised all references to conform fully to the journal’s formatting requirements.

5. Data availability should be clarified. The manuscript states that data are within the manuscript and supplementary files, but none are provided in the PDF. Either include them or revise the statement.

We thank the reviewer for this comment and have added the following clarification:

“The dataset used in this study is the property of the Qatar Ministry of Public Health (MOPH) and was provided to the researchers under a restricted-access agreement that prohibits sharing the dataset with third parties or making it publicly available. The data remain under restricted access to preserve patient confidentiality in compliance with national data privacy regulations. Researchers may request access to the dataset directly from the Ministry of Public Health via the Governmental Health Communication Center (https://www.moph.gov.qa/english/OurServices/eservices/Pages/Governmental-Health-Communication-Center.aspx). The raw data are therefore not publicly available, but aggregate and summary data are presented within the paper and its supplementary materials.”

Response to Reviewer 2:

1. Why choose 121 U/mL as the high/low cutoff point? Is there any literature to support this classification?

We thank the reviewer for this valuable comment. The 121 U/mL cutoff represented the median antibody titer among seropositive participants and was used as an internal, data-driven, exploratory stratification point to assess whether the magnitude of antibody response correlated with clinical outcomes such as COVID-19 infection or hospitalization. At the time of the study, no universally accepted or externally validated quantitative threshold existed to define protective immunity in kidney transplant recipients. Therefore, the median-based cutoff provided a pragmatic and unbiased means to explore these associations. This rationale has been clarified in the Methods section.

2. In the study, 96% of participants received the Pfizer vaccine, while only 4% received the Moderna vaccine. Could this imbalance affect the generalizability of the results? Is it possible to increase the sample size of Moderna vaccine recipients to further validate the conclusions?

Thank you for raising this point. Pfizer-BioNTech was the first available vaccine in Qatar, and kidney transplant recipients were prioritized for early vaccination among the general population. As a result, most of our patients received the Pfizer vaccine (96%). This study included all kidney transplant recipients who underwent antibody testing after receiving two vaccine doses; therefore, it was not possible to expand the Moderna subgroup. While vaccine type was included in both univariable and multivariable analyses and did not show statistically significant associations with outcomes, we have acknowledged this limitation in the Discussion section, noting that the small number of Moderna recipients may limit the generalizability of our findings to recipients of other mRNA vaccines.

3. In this study, the authors only detected the antibody titers once. It would be better to provide long-term data to show the relationship between antibody titer change and COVID-19 risk.

We thank the reviewer for this suggestion. We acknowledge that antibody titers were measured only once in our study, which limits the ability to assess longitudinal changes in antibody levels and their relationship with COVID-19 risk. This was due to the limited availability of the Elecsys® anti–SARS-CoV-2 S immunoassay in Qatar during the study period, which was prioritized for high-risk populations, including kidney transplant recipients. Despite this, our single post-vaccination measurement provides valuable insight into the association between antibody levels and COVID-19 incidence and severity in this population. We have now included this limitation in the Discussion section to clarify that long-term antibody kinetics could not be evaluated.

4. The authors only evaluated the humoral responses, should cell-mediated responses be included in this study to get more fully assess of the vaccine, also can be compared whether gender is the only one significant factor associated with poor responses.

We thank the reviewer for this important point. We agree that evaluating cell-mediated immunity, such as spike-specific CD4+ T-cell interferon-gamma responses, would provide a more comprehensive assessment of vaccine-induced immunity. However, due to the retrospective design of our study and the limitations of available clinical data, cellular immune responses were not assessed. We have now addressed this limitation in the revised Discussion. Additionally, we cite relevant literature on CD4+ T-cell responses in transplant recipients, highlighting gender-related differences in T-cell activity and the suppressive effects of anti-metabolite therapies, including mycophenolate mofetil and azathioprine, on both humoral and cellular immunity. This context supports our findings regarding gender and immunosuppressive regimen as significant factors associated with vaccine response.

5. The authors conclude that gender is the only factor associated with poor response, it would be better to further investigate the potential reasons.

We thank the reviewer for this observation. In the revised analysis, after performing multivariable regression, we identified additional factors associated with poor response to the COVID-19 mRNA vaccine beyond gender. Specifically, older age was also significantly associated with a higher risk of poor seroconversion. These findings are consistent with the concept of immunosenescence, where aging impairs both humoral and cellular immune responses. We have updated the Results and Discussion sections to reflect these additional factors and provide context regarding their biological plausibility.

6. Most participants received a third dose of the vaccine. but the article did not discuss its impact on subsequent infection rates or antibody levels. Could the authors analyze and discuss its effect on antibody titers and infection rates?

We thank the reviewer for this insightful comment. In our study, all antibody measurements were performed prior to administration of the third vaccine dose. Therefore, the impact of the third dose on subsequent antibody titers could not be directly evaluated. However, we did examine the uptake of the third dose across different patient groups (previous COVID-19 infection, seronegative, and seropositive), and found no significant differences between groups (P = 0.65).

Minor concern:

1. Lines 113-115: “125 had low antibody (anti-SARS-CoV-2 S protein114 RBD antibody titer < 121 U/mL and 125 had high antibody titers (anti-SARS-CoV-2 S protein115 RBD antibody titer > 121 U/mL), as shown in Figure 1”. It should be “≥ 121 U/mL”, need to add “= 121” this situation.

Thank you for your comment. This was corrected as suggested.

2. Lines 152-154: The manuscript is inconsistent in its use of SARS-CoV-2, some lack the dash between SARS and CoV, it needs to be consistent throughout the manuscript.

Thank you for your comment. This was corrected throughout the manuscript.

Attachment Submitted filename: Response to reviewers .docx

# Peer review: Decision Letter 1

**DOI:** 10.1371/journal.pone.0359640.r003

**Authors:** Engin Berber

22 Feb 2026

Dear Dr. Alkadi,

Thank you for submitting your manuscript to PLOS ONE. After careful consideration, we feel that it has merit but does not fully meet PLOS ONE’s publication criteria as it currently stands. Therefore, we invite you to submit a revised version of the manuscript that addresses the points raised during the review process.

==============================

The reviewers generally agree that the manuscript has been substantially improved and that prior concerns have been addressed. The study is considered technically sound, and the remaining issues are limited to clarifications, reporting refinements, and several interpretive/methodological points.

I am therefore inviting a **minor revision** . Please address the remaining reviewer comments, with particular attention to: (i) clarifying the timing of prior SARS-CoV-2 infection relative to vaccination and antibody assessment (or acknowledging if unavailable), (ii) clarifying the timing of third-dose vaccination relative to COVID-19 infection among those who developed infection, (iii) revising Table 2 to report individual p-values for immunosuppressive medication categories, (iv) clarifying cohort selection and generalizability, (v) revising use of the term “seroconversion” given the absence of baseline antibody testing, (vi) discussing possible confounding by unrecognized/subclinical infection, (vii) justifying the use of logistic regression instead of time-to-event analysis (and providing a sensitivity analysis if feasible), and (viii) replacing “vaccine efficacy” with “vaccine effectiveness” where appropriate.

Please provide a detailed point-by-point response and indicate all manuscript changes clearly in the tracked version.

==============================

Please submit your revised manuscript by Apr 08 2026 11:59PM. If you will need more time than this to complete your revisions, please reply to this message or contact the journal office at plosone@plos.org. When you're ready to submit your revision, log on to https://www.editorialmanager.com/pone/ and select the 'Submissions Needing Revision' folder to locate your manuscript file.

- A letter that responds to each point raised by the academic editor and reviewer(s). You should upload this letter as a separate file labeled 'Response to Reviewers'.
- A marked-up copy of your manuscript that highlights changes made to the original version. You should upload this as a separate file labeled 'Revised Manuscript with Track Changes'.
- An unmarked version of your revised paper without tracked changes. You should upload this as a separate file labeled 'Manuscript'.

If applicable, we recommend that you deposit your laboratory protocols in protocols.io to enhance the reproducibility of your results. Protocols.io assigns your protocol its own identifier (DOI) so that it can be cited independently in the future. For instructions see: https://journals.plos.org/plosone/s/submission-guidelines#loc-laboratory-protocols. Additionally, PLOS ONE offers an option for publishing peer-reviewed Lab Protocol articles, which describe protocols hosted on protocols.io. Read more information on sharing protocols at https://plos.org/protocols?utm_medium=editorial-email&utm_source=authorletters&utm_campaign=protocols.

We look forward to receiving your revised manuscript.

Kind regards,

Engin Berber, D.V.M., Ph.D.

Academic Editor

PLOS One

Journal Requirements:

If the reviewer comments include a recommendation to cite specific previously published works, please review and evaluate these publications to determine whether they are relevant and should be cited. There is no requirement to cite these works unless the editor has indicated otherwise.

Please review your reference list to ensure that it is complete and correct. If you have cited papers that have been retracted, please include the rationale for doing so in the manuscript text, or remove these references and replace them with relevant current references. Any changes to the reference list should be mentioned in the rebuttal letter that accompanies your revised manuscript. If you need to cite a retracted article, indicate the article’s retracted status in the References list and also include a citation and full reference for the retraction notice.

[Note: HTML markup is below. Please do not edit.]

Reviewers' comments:

Reviewer's Responses to Questions

**Comments to the Author**

Reviewer #2: All comments have been addressed

Reviewer #3: All comments have been addressed

Reviewer #4: (No Response)

**********

2. Is the manuscript technically sound, and do the data support the conclusions?

Reviewer #2: Yes

Reviewer #3: Yes

Reviewer #4: Yes

**********

3. Has the statistical analysis been performed appropriately and rigorously?

Reviewer #2: Yes

Reviewer #3: Yes

Reviewer #4: I Don't Know

**********

4. Have the authors made all data underlying the findings in their manuscript fully available?

The PLOS Data policy

Reviewer #2: Yes

Reviewer #3: Yes

Reviewer #4: Yes

**********

5. Is the manuscript presented in an intelligible fashion and written in standard English?

Reviewer #2: Yes

Reviewer #3: Yes

Reviewer #4: Yes

**********

Reviewer #2: The authors have fully addressed all of my major and minor concerns. The revisions substantially improved the scientific rigor, clarity, and completeness of the manuscript.

I still have one more concern about “Does the timing of prior infection affect antibody titers?”

In the study, 45 patients with a history of previous infection had a median antibody titer of 250 U/mL (the upper limit of detection). Combining all past infections into a single group does not allow for definitive the time of infection (pre-vaccination? after first dose? after second dose?) But infections occurring before vaccination or after vaccination have entirely different biological significance for antibody maturation and memory response. It would be helpful if the authors could further clarify this aspect.

Reviewer #3: This paper assessed the antibody level and clinical outcomes of 2-dose series of mRNA vaccine among kidney transplant recipients. The paper is well-written. The data presentation is clear and thorough after addressing comments from the previous reviewers.

Specific comments:

1. The timing of administration of the third dose of vaccine in relation to COVID-19 infection. Taking a booster dose may reduce the risk of infection, and not all recipients who were eventually infected with COVID-19 had received a third dose prior to infection. Therefore, comparison of infection rate may be biased by the timing of third dose (i.e. before third dose, after third dose, or never had third dose). The authors could consider adding a few sentences or tables rows in the results section to show the distribution of timing of third dose among recipients who were infected with COVID-19.

2. In Table 2, there should be 6 individual p-values reported for immunosuppression medications rather than a single p-value, as these categories are not mutually exclusive.

Reviewer #4: This study examined seroconversion rates among kidney transplant recipients following COVID-19 mRNA vaccination and identified factors associated with immunogenicity. I believe the authors have adequately addressed the concerns raised by the prior reviewers.

If the editors feel it is appropriate, I have several comments for consideration:

The study included 372 kidney transplant recipients who received two doses of a COVID-19 mRNA vaccine and had anti–SARS-CoV-2 spike protein receptor-binding domain antibodies measured at least 14 days after the second dose and before receipt of a third dose. An important question is what proportion of all vaccinated kidney transplant recipients met this definition. If this represents a small subset, were there differences between recipients who qualified for inclusion and those who did not? This could affect the generalizability of the study’s findings to the broader kidney transplant population.

In Line 145, the authors state: “Of the remaining 327 patients, 250 (76%) had seroconversion.” Because baseline antibody levels were not measured, how can the authors be certain that positive antibody results represent true seroconversion? Is it possible that some patients had detectable antibodies prior to vaccination due to previously unrecognized SARS-CoV-2 infection?

Relatedly, can the authors comment on the possibility of subclinical COVID-19 infection prior to the emergence of the Omicron variant? Pre-Omicron variants would have been circulating during the interval between the second vaccine dose and antibody assessment. The comparisons between seropositive versus seronegative groups, as well as low versus high antibody-level groups, would be more robust if subclinical infection were unlikely during this period. Otherwise, some individuals categorized as seropositive or having high antibody levels may have had antibody responses attributable to prior infection rather than vaccination, which could confound the observed protective associations.

It appears that time-to-COVID-19 event data were available. If so, Cox proportional hazards regression might be a more appropriate analytical approach than logistic regression. Is there a reason logistic regression was chosen instead of a time-to-event analysis?

Minor comment: In Line 330, the term “vaccine effectiveness” should be used instead of “vaccine efficacy,” as the latter is more appropriately reserved for randomized controlled trials.

**********

what does this mean?). If published, this will include your full peer review and any attached files.

If you choose “no”, your identity will remain anonymous but your review may still be made public.

**Do you want your identity to be public for this peer review?**  For information about this choice, including consent withdrawal, please see our Privacy Policy

Reviewer #2: No

Reviewer #3: No

Reviewer #4: **Yes:**  Nabin K. Shrestha

**********

[NOTE: If reviewer comments were submitted as an attachment file, they will be attached to this email and accessible via the submission site. Please log into your account, locate the manuscript record, and check for the action link "View Attachments". If this link does not appear, there are no attachment files.]

To ensure your figures meet our technical requirements, please review our figure guidelines: https://journals.plos.org/plosone/s/figures

You may also use PLOS’s free figure tool, NAAS, to help you prepare publication quality figures: https://journals.plos.org/plosone/s/figures#loc-tools-for-figure-preparation.

NAAS will assess whether your figures meet our technical requirements by comparing each figure against our figure specifications.

# Author comment: Author response to Decision Letter 2

**DOI:** 10.1371/journal.pone.0359640.r004

8 Apr 2026

We sincerely thank the reviewers and the editor for their careful evaluation of our manuscript and for their constructive comments. We are pleased that the reviewers consider the study technically sound and recognize the substantial improvements made in the previous revision.

We have carefully addressed all remaining comments, with particular attention to clarifying methodological details, refining reporting, and enhancing interpretation where requested. These revisions have further strengthened the clarity, transparency, and overall quality of the manuscript.

Below, we provide a detailed, point-by-point response to each comment and indicate the corresponding revisions made in the manuscript. All changes have been clearly marked in the revised version.

Response to Academic Editor:

1) Clarifying the timing of prior SARS-CoV-2 infection relative to vaccination and antibody assessment (or acknowledging if unavailable).

Thank you for your comment. This was clarified in the revised manuscript as follows (lines 179-191): Forty-five patients had a previous COVID-19 infection before anti‑SARS‑CoV‑2 S immunoassay testing. Of these, 21 patients had COVID-19 infection before vaccination, while 24 patients had infection after vaccination (6 patients developed infection after the first vaccine dose but before the second dose, and 18 patients developed infection after the second dose). The median time from infection to antibody testing was 381 days (IQR 289–421) for infections before vaccination, 112 days (IQR 99–131) for infections after the first dose, and 88.5 days (IQR 65.5–110.8) for infections after the second dose. The interval differed significantly across groups (p < 0.001). Despite these differences in timing, comparison of antibody levels across the three groups (infection before vaccination, infection after the first dose but before the second dose, and infection after the second dose) showed no statistically significant difference in antibody titers (median antibody level of 250 U/mL in all groups, P = 0.54). In addition, Spearman correlation analysis demonstrated no significant association between the time from infection to antibody testing and antibody titers (ρ = −0.064, P = 0.68).

2) Clarifying the timing of third-dose vaccination relative to COVID-19 infection among those who developed infection.

Thank you for your comment. This was clarified in the revised manuscript as follows (lines 222-244): During the follow-up period after anti‑SARS‑CoV‑2 S immunoassay testing, 74% of patients received a third COVID-19 mRNA vaccine (n=275), with no difference across groups (P = 0.65). Among patients with previous infection, 6 developed COVID-19 infection (3 after the third vaccine dose and 3 who did not receive the third dose). In seropositive patients, 66 patients developed infection (1 before the third dose, 38 after the third dose, and 27 who did not receive the third dose). In seronegative patients, 28 developed infection (22 after the third dose and 6 who did not receive the third dose). The time between the third vaccine dose and subsequent COVID-19 infection differed significantly between patients with previous infection and seronegative patients (median 118 vs 132 days, P = 0.04). Differences between previous infection and seropositive patients (118 versus 128 days, P = 0.14) and between seronegative and seropositive patients (128 versus 132 days, P = 0.06) were not statistically significant.

We also have added a row in Table 1 showing the distribution of the timing of the third vaccine dose among recipients who were infected with COVID-19.

3) Revising Table 2 to report individual p-values for immunosuppressive medication categories

Thank you for your comment. In the revised manuscript, we have included individual p-values for each immunosuppressive medication category in Table 2.

4) Clarifying cohort selection and generalizability.

Thank you for your comment. This was clarified in the revised manuscript as follows (lines 89-93): This was a retrospective cohort study of adult kidney transplant recipients who received two doses of COVID-19 mRNA vaccine after transplantation and had their anti‑SARS‑CoV‑2 S protein RBD antibodies measured at least 14 days after the second dose and before receiving a third dose. These criteria were established prior to testing and all patients meeting them who were tested before October 1, 2021, were included and followed until February 1, 2022.

Because all eligible patients were included consecutively, we believe the risk of selection bias was minimal in the study.

5) Revising use of the term “seroconversion” given the absence of baseline antibody testing.

Thank you for your comment. We agree that the absence of baseline antibody testing limits the ability to accurately define true seroconversion. Our goal was to identify patients with detectable antibodies after vaccination and those without detectable antibodies after vaccination. Therefore, we have revised the terminology throughout the manuscript and replaced “seroconversion” with “antibody status” or “seropositivity”.

6) Discussing possible confounding by unrecognized/subclinical infection.

Thank you for your comment. We have addressed this in the revised Discussion by adding the following statement (lines 42-427): We also cannot completely rule out the possibility that some patients had unrecognized or subclinical SARS-CoV-2 infection prior to antibody measurement, including infections with pre-Omicron variants. As a result, comparisons between seropositive versus seronegative patients, or between those with low versus high antibody levels, may be affected, since some individuals categorized as seropositive or having high antibody levels could have had antibody responses attributable to prior infection rather than vaccination.

7) Justifying the use of logistic regression instead of time-to-event analysis (and providing a sensitivity analysis if feasible)

Thank you for your comment. Logistic regression was used because the primary study outcomes—poor vaccine response (seronegativity) and high versus low antibody titers— were binary variables derived from a single antibody measurement obtained following the second vaccine dose, and the primary objective was to assess associations with antibody response. Although post-vaccination COVID-19 infections during follow-up were recorded, a time-to-event analysis using Cox proportional hazards regression was not performed, as this would have introduced several methodological complexities beyond the scope of the present study, including the need to incorporate the third vaccine dose as a time-varying exposure and to account for potential competing events—most notably death—neither of which were adequately captured in the available dataset. We have clarified this rationale in the Methods section of the revised manuscript (lines 161-169).

8) Replacing “vaccine efficacy” with “vaccine effectiveness” where appropriate.

Thank you for your comment. We have replaced “vaccine efficacy” with “vaccine effectiveness” throughout the manuscript to reflect the observational nature of our study.

Response to Reviewer 2:

1) Does the timing of prior infection affect antibody titers? In the study, 45 patients with a history of previous infection had a median antibody titer of 250 U/mL (the upper limit of detection). Combining all past infections into a single group does not allow for definitive the time of infection (pre-vaccination? after first dose? after second dose?) But infections occurring before vaccination or after vaccination have entirely different biological significance for antibody maturation and memory response. It would be helpful if the authors could further clarify this aspect.

Thank you for this important and insightful comment. We agree that the timing of prior infection in relation to vaccination may have different biological implications for antibody maturation and memory response. This was clarified in the revised manuscript as follows (lines 179-191): Forty-five patients had a previous COVID-19 infection before anti‑SARS‑CoV‑2 S immunoassay testing. Of these, 21 patients had COVID-19 infection before vaccination, while 24 patients had infection after vaccination (6 patients developed infection after the first vaccine dose but before the second dose, and 18 patients developed infection after the second dose). The median time from infection to antibody testing was 381 days (IQR 289–421) for infections before vaccination, 112 days (IQR 99–131) for infections after the first dose, and 88.5 days (IQR 65.5–110.8) for infections after the second dose. The interval differed significantly across groups (p < 0.001). Despite these differences in timing, comparison of antibody levels across the three groups (infection before vaccination, infection after the first dose but before the second dose, and infection after the second dose) showed no statistically significant difference in antibody titers (median antibody level of 250 U/mL in all groups, P = 0.54). In addition, Spearman correlation analysis demonstrated no significant association between the time from infection to antibody testing and antibody titers (ρ = −0.064, P = 0.68).

Response to Reviewer 3:

1) The timing of administration of the third dose of vaccine in relation to COVID-19 infection. Taking a booster dose may reduce the risk of infection, and not all recipients who were eventually infected with COVID-19 had received a third dose prior to infection. Therefore, comparison of infection rate may be biased by the timing of third dose (i.e. before third dose, after third dose, or never had third dose). The authors could consider adding a few sentences or tables rows in the results section to show the distribution of timing of third dose among recipients who were infected with COVID-19.

Thank you for your comment. This was clarified in the revised manuscript as follows (lines 222-244): During the follow-up period after anti‑SARS‑CoV‑2 S immunoassay testing, 74% of patients received a third COVID-19 mRNA vaccine (n=275), with no difference across groups (P = 0.65). Among patients with previous infection, 6 developed COVID-19 infection (3 after the third vaccine dose and 3 who did not receive the third dose). In seropositive patients, 66 patients developed infection (1 before the third dose, 38 after the third dose, and 27 who did not receive the third dose). In seronegative patients, 28 developed infection (22 after the third dose and 6 who did not receive the third dose). The time between the third vaccine dose and subsequent COVID-19 infection differed significantly between patients with previous infection and seronegative patients (median 118 vs 132 days, P = 0.04). Differences between previous infection and seropositive patients (118 versus 128 days, P = 0.14) and between seronegative and seropositive patients (128 versus 132 days, P = 0.06) were not statistically significant.

We also have added a row in Table 1 showing the distribution of the timing of the third vaccine dose among recipients who were infected with COVID-19.

2) In Table 2, there should be 6 individual p-values reported for immunosuppression medications rather than a single p-value, as these categories are not mutually exclusive.

Thank you for your comment. In the revised manuscript, we have included individual p-values for each immunosuppressive medication category in Table 2.

Response to Reviewer 4:

1) The study included 372 kidney transplant recipients who received two doses of a COVID-19 mRNA vaccine and had anti–SARS-CoV-2 spike protein receptor-binding domain antibodies measured at least 14 days after the second dose and before receipt of a third dose. An important question is what proportion of all vaccinated kidney transplant recipients met this definition. If this represents a small subset, were there differences between recipients who qualified for inclusion and those who did not? This could affect the generalizability of the study’s findings to the broader kidney transplant population.

Thank you for your comment. This was clarified in the revised manuscript as follows (lines 89-93): This was a retrospective cohort study of adult kidney transplant recipients who received two doses of COVID-19 mRNA vaccine after transplantation and had their anti‑SARS‑CoV‑2 S protein RBD antibodies measured at least 14 days after the second dose and before receiving a third dose. These criteria were established prior to testing and all patients meeting them who were tested before October 1, 2021, were included and followed until February 1, 2022.

Because all eligible patients were included consecutively, we believe the risk of selection bias was minimal in the study.

2) In Line 145, the authors state: “Of the remaining 327 patients, 250 (76%) had seroconversion.” Because baseline antibody levels were not measured, how can the authors be certain that positive antibody results represent true seroconversion? Is it possible that some patients had detectable antibodies prior to vaccination due to previously unrecognized SARS-CoV-2 infection?

Thank you for your comment. We agree that the absence of baseline antibody testing limits the ability to accurately define true seroconversion. Our goal was to identify patients with detectable antibodies after vaccination and those without detectable antibodies after vaccination. Therefore, we have revised the terminology throughout the manuscript and replaced “seroconversion” with “antibody status” or “seropositivity”.

3) Relatedly, can the authors comment on the possibility of subclinical COVID-19 infection prior to the emergence of the Omicron variant? Pre-Omicron variants would have been circulating during the interval between the second vaccine dose and antibody assessment. The comparisons between seropositive versus seronegative groups, as well as low versus high antibody-level groups, would be more robust if subclinical infection were unlikely during this period. Otherwise, some individuals categorized as seropositive or having high antibody levels may have had antibody responses attributable to prior infection rather than vaccination, which could confound the observed protective associations.

Thank you for your comment. We have addressed this in the revised Discussion by adding the following statement (lines 422-427): We also cannot completely rule out the possibility that some patients had unrecognized or subclinical SARS-CoV-2 infection prior to antibody measurement, including infections with pre-Omicron variants. As a result, comparisons between seropositive versus seronegative patients, or between those with low versus high antibody levels, may be affected, since some individuals categorized as seropositive or having high antibody levels could have had antibody responses attributable to prior infection rather than vaccination.

4) It appears that time-to-COVID-19 event data were available. If so, Cox proportional hazards regression might be a more appropriate analytical approach than logistic regression. Is there a reason logistic regression was chosen instead of a time-to-event analysis?

Thank you for your comment. Logistic regression was used because the primary study outcomes—poor vaccine response (seronegativity) and high versus low antibody titers— were binary variables derived from a single antibody measurement obtained following the second vaccine dose, and the primary objective was to assess associations with antibody response. Although post-vaccination COVID-19 infections during follow-up were recorded, a time-to-event analysis using Cox proportional hazards regression was not performed, as this would have introduced several methodological complexities beyond the scope of the present study, including the need to incorporate the third vaccine dose as a time-varying exposure and to account for potential competing events—most notably death—neither of which were adequately captured in the available dataset. We have clarified this rationale in the Methods section of the revised manuscript (lines 161-169).

5) In Line 330, the term “vaccine effectiveness” should be used instead of “vaccine efficacy,” as the latter is more appropriately reserved for randomized controlled trials.

Thank you for your comment. We have replaced “vaccine efficacy” with “vaccine effectiveness” throughout the manuscript to reflect the observational nature of our study.

Attachment Submitted filename: Response to reviewers (R2).docx

# Peer review: Decision Letter 2

**DOI:** 10.1371/journal.pone.0359640.r005

**Authors:** Engin Berber

3 May 2026

Dear Dr. Alkadi,

Thank you for submitting your manuscript to PLOS ONE. After careful consideration, we feel that it has merit but does not fully meet PLOS ONE’s publication criteria as it currently stands. Therefore, we invite you to submit a revised version of the manuscript that addresses the points raised during the review process.

Please submit your revised manuscript by Jun 17 2026 11:59PM. If you will need more time than this to complete your revisions, please reply to this message or contact the journal office at plosone@plos.org. When you're ready to submit your revision, log on to https://www.editorialmanager.com/pone/ and select the 'Submissions Needing Revision' folder to locate your manuscript file.

- A letter that responds to each point raised by the academic editor and reviewer(s). You should upload this letter as a separate file labeled 'Response to Reviewers'.
- A marked-up copy of your manuscript that highlights changes made to the original version. You should upload this as a separate file labeled 'Revised Manuscript with Track Changes'.
- An unmarked version of your revised paper without tracked changes. You should upload this as a separate file labeled 'Manuscript'.

If you would like to make changes to your financial disclosure, please include your updated statement in your cover letter. Guidelines for resubmitting your figure files are available below the reviewer comments at the end of this letter.

If applicable, we recommend that you deposit your laboratory protocols in protocols.io to enhance the reproducibility of your results. Protocols.io assigns your protocol its own identifier (DOI) so that it can be cited independently in the future. For instructions see: https://journals.plos.org/plosone/s/submission-guidelines#loc-laboratory-protocols. Additionally, PLOS ONE offers an option for publishing peer-reviewed Lab Protocol articles, which describe protocols hosted on protocols.io. Read more information on sharing protocols at https://plos.org/protocols?utm_medium=editorial-email&utm_source=authorletters&utm_campaign=protocols.

As the corresponding author, your ORCID iD is verified in the submission system and will appear in the published article. PLOS supports the use of ORCID, and we encourage all coauthors to register for an ORCID iD and use it as well. Please encourage your coauthors to verify their ORCID iD within the submission system before final acceptance, as unverified ORCID iDs will not appear in the published article. *Only*  the individual author can complete the verification step; PLOS staff *cannot*  verify ORCID iDs on behalf of authors.

We look forward to receiving your revised manuscript.

Kind regards,

Engin Berber, D.V.M., Ph.D.

Academic Editor

PLOS One

Journal Requirements:

If the reviewer comments include a recommendation to cite specific previously published works, please review and evaluate these publications to determine whether they are relevant and should be cited. There is no requirement to cite these works unless the editor has indicated otherwise.

Please review your reference list to ensure that it is complete and correct. If you have cited papers that have been retracted, please include the rationale for doing so in the manuscript text, or remove these references and replace them with relevant current references. Any changes to the reference list should be mentioned in the rebuttal letter that accompanies your revised manuscript. If you need to cite a retracted article, indicate the article’s retracted status in the References list and also include a citation and full reference for the retraction notice.

Additional Editor Comments:

Please address concerns raised by Reviewer 4.

[Note: HTML markup is below. Please do not edit.]

Reviewers' comments:

Reviewer's Responses to Questions

**Comments to the Author**

Reviewer #2: All comments have been addressed

Reviewer #3: All comments have been addressed

Reviewer #4: (No Response)

**********

2. Is the manuscript technically sound, and do the data support the conclusions?

Reviewer #2: Yes

Reviewer #3: Yes

Reviewer #4: Yes

**********

3. Has the statistical analysis been performed appropriately and rigorously?

Reviewer #2: Yes

Reviewer #3: Yes

Reviewer #4: Yes

**********

4. Have the authors made all data underlying the findings in their manuscript fully available?

The PLOS Data policy

Reviewer #2: Yes

Reviewer #3: No

Reviewer #4: Yes

**********

5. Is the manuscript presented in an intelligible fashion and written in standard English?

Reviewer #2: Yes

Reviewer #3: Yes

Reviewer #4: Yes

**********

Reviewer #2: No further comments.

The authors have addressed the core concerns raised in my initial review The revision is improved, with greater clarity regarding the study's aims, limitations, and the need for future validation.

Reviewer #3: (No Response)

Reviewer #4: The question about generalizability has not been adequately answered. The framing of the question may have been inadequate.

This study included kidney transplant recipients who had spike protein antibody levels checked before their third COVID-19 vaccine dose. It was not standard practice to check spike protein antibodies before a third COVID-19 mrRNA vaccine was given. Since this was a retrospective study, one could not go back in time and check anti-SARS_CoV-2 spike protein antibodies in patients in whom these antibodies were not checked. Unless there was a national or an institutional policy not to administer a third dose of vaccine until spike antibody levels were checked, there would surely have been patients who did not have spike antibody levels tested before their third vaccine dose. So the question is what proportion of kidney transplant recipients had spike protein antibody levels checked between their second and third dose? Were these people systematically different from those who did not get such testing done? Was there something about these people that prompted spike antibody level testing before their third vaccine dose? If so, there may be questions about generalizability.

The fact that all "eligible" kidney transplant patients were included consecutively does not necessarily mean the study can be generalized to the broader kidney transplant population, which would include "ineligible" kidney transplant recipients, that is those who did not have spike antibody testing prior to receiving a third dose of vaccine.

**********

what does this mean?). If published, this will include your full peer review and any attached files.

If you choose “no”, your identity will remain anonymous but your review may still be made public.

**Do you want your identity to be public for this peer review?**  For information about this choice, including consent withdrawal, please see our Privacy Policy

Reviewer #2: No

Reviewer #3: No

Reviewer #4: **Yes:**  Nabin K. Shrestha

**********

[NOTE: If reviewer comments were submitted as an attachment file, they will be attached to this email and accessible via the submission site. Please log into your account, locate the manuscript record, and check for the action link "View Attachments". If this link does not appear, there are no attachment files.]

To ensure your figures meet our technical requirements, please review our figure guidelines: https://journals.plos.org/plosone/s/figures

You may also use PLOS’s free figure tool, NAAS, to help you prepare publication quality figures: https://journals.plos.org/plosone/s/figures#loc-tools-for-figure-preparation.

NAAS will assess whether your figures meet our technical requirements by comparing each figure against our figure specifications.

# Author comment: Author response to Decision Letter 3

**DOI:** 10.1371/journal.pone.0359640.r006

6 Jun 2026

We sincerely thank the reviewer for this important and insightful comment regarding the potential for selection bias and generalizability. As correctly noted, it was not standard clinical practice to measure spike protein antibody levels prior to administration of a third COVID-19 mRNA vaccine dose, and there was no national or institutional policy requiring antibody testing before booster vaccination. We also acknowledge that inclusion of "eligible" patients does not automatically ensure generalizability to the broader kidney transplant population, which includes patients who did not undergo antibody testing prior to receiving a third dose.

Regarding the proportion of kidney transplant recipients who underwent spike protein antibody testing performed between the second and third vaccine doses, the anti-SARS-CoV-2 S protein RBD immunoassay was available in Qatar in limited quantities between June and September 2021. In addition, the national COVID-19 booster vaccination program was initiated on 24 August 2021.

Overall, 372 of 577 eligible adult kidney transplant recipients (64%) underwent anti-SARS CoV 2 S protein RBD antibody testing after receiving two doses of the COVID-19 mRNA vaccine and prior to receipt of the third dose. Baseline characteristics were comparable between tested and untested eligible patients, with no statistically significant differences in age, gender, race, or year of transplantation, suggesting that selection bias is unlikely to have significantly affected the findings (Supplementary Table 1).

This point has been clarified in the revised manuscript, including the Methods, Results, and Discussion (limitations) sections (Lines 87-93; 152-158; 374-378) and Figure 1.

Attachment Submitted filename: Response to reviewers (R3).docx

# Editor report: Decision Letter 3

**DOI:** 10.1371/journal.pone.0359640.r007

**Authors:** Engin Berber

17 Jul 2026

Dear Dr. Alkadi,

Thank you for submitting your manuscript to PLOS ONE. After careful consideration, we feel that it has merit but does not fully meet PLOS ONE’s publication criteria as it currently stands. Therefore, we invite you to submit a revised version of the manuscript that addresses the points raised during the review process.

==============================

The additional information concerning cohort selection has substantially addressed the remaining reviewer concern. In particular, reporting that 372 of 577 eligible kidney transplant recipients underwent antibody testing, adding the untested recipients to the study flow diagram, and comparing selected baseline characteristics between tested and untested patients have improved the transparency of the study. During final editorial review, several statistical-reporting inconsistencies and interpretive issues are detected and must be resolved before our final decision.

Please carefully verify the numerical values and P values in the Results and tables, correct the statistical-test description in Table 1, and clarify the analytical method used for the time-to-infection analysis presented in Figure 2.

The manuscript should also distinguish associations from prediction or vaccine effectiveness. Because most participants received a third vaccine dose during follow-up and this exposure was not incorporated into an adjusted time-to-event model, the subsequent infection findings should be presented as exploratory, unadjusted associations. Statements regarding neutralizing-antibody effectiveness, confirmed protection against Omicron, prediction of disease severity, and antibody-guided booster administration should therefore be removed or appropriately qualified. Please also moderate the conclusion regarding selection bias, acknowledge the effect of the assay’s upper measurement limit on analyses involving previously infected recipients, define the criteria used to diagnose COVID-19 and disease severity, and correct the numerical and language inconsistencies identified in the manuscript. Please see Editorial comments in detail below.

Sincerely,

==============================

Please submit your revised manuscript by Aug 31 2026 11:59PM. If you will need more time than this to complete your revisions, please reply to this message or contact the journal office at plosone@plos.org. When you're ready to submit your revision, log on to https://www.editorialmanager.com/pone/ and select the 'Submissions Needing Revision' folder to locate your manuscript file.

- A letter that responds to each point raised by the academic editor and reviewer(s). You should upload this letter as a separate file labeled 'Response to Reviewers'.
- A marked-up copy of your manuscript that highlights changes made to the original version. You should upload this as a separate file labeled 'Revised Manuscript with Track Changes'.
- An unmarked version of your revised paper without tracked changes. You should upload this as a separate file labeled 'Manuscript'.

If you would like to make changes to your financial disclosure, please include your updated statement in your cover letter. Guidelines for resubmitting your figure files are available below the reviewer comments at the end of this letter.

If applicable, we recommend that you deposit your laboratory protocols in protocols.io to enhance the reproducibility of your results. Protocols.io assigns your protocol its own identifier (DOI) so that it can be cited independently in the future. For instructions see: https://journals.plos.org/plosone/s/submission-guidelines#loc-laboratory-protocols. Additionally, PLOS ONE offers an option for publishing peer-reviewed Lab Protocol articles, which describe protocols hosted on protocols.io. Read more information on sharing protocols at https://plos.org/protocols?utm_medium=editorial-email&utm_source=authorletters&utm_campaign=protocols.

As the corresponding author, your ORCID iD is verified in the submission system and will appear in the published article. PLOS supports the use of ORCID, and we encourage all coauthors to register for an ORCID iD and use it as well. Please encourage your coauthors to verify their ORCID iD within the submission system before final acceptance, as unverified ORCID iDs will not appear in the published article. *Only*  the individual author can complete the verification step; PLOS staff *cannot*  verify ORCID iDs on behalf of authors.

We look forward to receiving your revised manuscript.

Kind regards,

Engin Berber, D.V.M., Ph.D.

Academic Editor

PLOS One

Journal Requirements:

1. If the reviewer comments include a recommendation to cite specific previously published works, please review and evaluate these publications to determine whether they are relevant and should be cited. There is no requirement to cite these works unless the editor has indicated otherwise.

2. Please review your reference list to ensure that it is complete and correct. If you have cited papers that have been retracted, please include the rationale for doing so in the manuscript text, or remove these references and replace them with relevant current references. Any changes to the reference list should be mentioned in the rebuttal letter that accompanies your revised manuscript. If you need to cite a retracted article, indicate the article’s retracted status in the References list and also include a citation and full reference for the retraction notice.

Additional Editor Comments:

The Methods state that Cox regression was not performed, but Section 3.5 and Figure 2 nevertheless report “time to infection” comparisons and a P value of 0.007. The statistical procedure is not identified. The authors must state: Whether Kaplan–Meier analysis and a log-rank test were used. How participants without infection were censored. The censoring date and whether death, loss to follow-up, or third-dose vaccination affected censoring. Whether the reported P value is an overall test or a pairwise comparison. Whether pairwise comparisons were adjusted for multiple testing. These details are required for Section 3.5 and Figure 2.

The manuscript states that antibody levels were evaluated for their ability to “predict” infection and severity. However, the comparisons of subsequent infection are primarily unadjusted proportions. Moreover, 74% of participants received a third vaccine dose during follow-up, and the third dose was not incorporated as a time-varying exposure. Consequently, the study demonstrates an **association** , not independent prediction, vaccine effectiveness, or causation.

The authors should: Replace “predicting the incidence and severity” with “examining associations with subsequent infection and hospitalization.” Avoid stating that binding antibodies “remained effective against Omicron.” Avoid referring to the measured antibodies as neutralizing antibodies because neutralization was not measured. Remove or substantially soften the recommendation to test antibodies at 4–6 months and administer boosters according to the result. This should be presented only as a hypothesis requiring prospective evaluation. Replace “hospitalization for severe disease” unless a validated definition of severe COVID-19 was applied.

The response to Reviewer 4 is substantially satisfactory, but similarity in age, sex, geographic/racial category, and transplant year does not eliminate selection bias. Clinical factors such as immunosuppressive regimen, renal function, comorbidities, healthcare utilization, or clinician reasons for ordering testing may still differ. The limitation should read: “The similarity of the measured baseline characteristics reduces concern regarding selection bias; however, residual selection bias related to unmeasured clinical characteristics or reasons for antibody testing cannot be excluded.”

Define how COVID-19 infection was diagnosed—PCR, antigen testing, clinical diagnosis, or another criterion.

Explain how infections were attributed to Omicron, or revise the text to “infections occurring during the Omicron-predominant period.”

Include the informed-consent waiver in the submission-system ethics statement, consistent with the Methods.

Correct language errors including “antibodyd,” “anit-SARS-CoV-2,” “A Poor response,” and inconsistent capitalization.

Several errors need direct verification against the source dataset:

**Lines 213–214:**  The manuscript says female sex was more common in the seronegative group “75% versus 62%.” Those are the reported male percentages and are also presented in the reverse order. Based on Table 2, female percentages appear to be **38% versus 25%** .

**Lines 240–242:**  Diabetes is described as “69% versus 53%,” but Table 4 reports **69 patients (55%) versus 53 patients (42%)** .

**Lines 243–244:**  The text gives P = 0.02 for the interval between dose 2 and antibody testing, while Table 4 gives **P = 0.002** .

**Table 4:**  “Months from transplant” is reported as 86.8 versus 86.9 months with P = 0.02, while Table 5 reports no association after median categorization. The P value should be checked and the significant result discussed if correct.

**Table 1 footnote:**  It incorrectly states that one-way ANOVA was used for categorical variables. Categorical comparisons should be identified as chi-square or Fisher’s exact tests. The authors should identify precisely whether ANOVA or Kruskal–Wallis testing was used for each continuous variable.

[Note: HTML markup is below. Please do not edit.]

[NOTE: If reviewer comments were submitted as an attachment file, they will be attached to this email and accessible via the submission site. Please log into your account, locate the manuscript record, and check for the action link "View Attachments". If this link does not appear, there are no attachment files.]

To ensure your figures meet our technical requirements, please review our figure guidelines: https://journals.plos.org/plosone/s/figures

You may also use PLOS’s free figure tool, NAAS, to help you prepare publication quality figures: https://journals.plos.org/plosone/s/figures#loc-tools-for-figure-preparation.

NAAS will assess whether your figures meet our technical requirements by comparing each figure against our figure specifications.

# Author comment: Author response to Decision Letter 4

**DOI:** 10.1371/journal.pone.0359640.r008

9 Sep 2026

Dear Dr. Berber,

Thank you for your careful review of our manuscript and for the opportunity to submit a revised version. We appreciate your detailed comments and recommendations, which helped us improve the statistical reporting, clarify the interpretation of our findings, and improve the overall consistency of the manuscript.

We have carefully addressed each of the editorial comments and revised the manuscript accordingly. Below, we provide a detailed, point-by-point response to each comment and indicate the corresponding revisions made in the manuscript. All changes have been clearly marked in the revised version.

Response to Additional Editor Comments:

1) The Methods state that Cox regression was not performed, but Section 3.5 and Figure 2 nevertheless report “time to infection” comparisons and a P value of 0.007. The statistical procedure is not identified. The authors must state: Whether Kaplan–Meier analysis and a log-rank test were used. How participants without infection were censored. The censoring date and whether death, loss to follow-up, or third-dose vaccination affected censoring. Whether the reported P value is an overall test or a pairwise comparison. Whether pairwise comparisons were adjusted for multiple testing. These details are required for Section 3.5 and Figure 2.

Thank you for your valuable comment. We have revised Section 2.4 (Statistical analysis) of the Materials and Methods and Section 3.5 (Time to COVID-19 infection) of the Results to explicitly describe the time-to-infection analysis. Time to COVID-19 infection was analyzed using Kaplan–Meier survival analysis, with the date of the anti-SARS-CoV-2 S immunoassay test as time zero and subsequent COVID-19 infection as the event. Participants who did not develop COVID-19 infection were censored on February 1, 2022. No participants were censored because of death, loss to follow-up, or receipt of a third vaccine dose. The Kaplan–Meier curves for the four groups were compared using an overall log-rank test. After verification and reanalysis of the data, the overall log-rank test yielded P = 0.021; accordingly, the previously reported P value of 0.007 has been corrected. No pairwise log-rank comparisons were performed; therefore, no adjustment for multiple comparisons was required. Figure 2, including the figure legend, has been revised accordingly.

2) The manuscript states that antibody levels were evaluated for their ability to “predict” infection and severity. However, the comparisons of subsequent infection are primarily unadjusted proportions. Moreover, 74% of participants received a third vaccine dose during follow-up, and the third dose was not incorporated as a time-varying exposure. Consequently, the study demonstrates an association, not independent prediction, vaccine effectiveness, or causation. The authors should: Replace “predicting the incidence and severity” with “examining associations with subsequent infection and hospitalization.” Avoid stating that binding antibodies “remained effective against Omicron.” Avoid referring to the measured antibodies as neutralizing antibodies because neutralization was not measured. Remove or substantially soften the recommendation to test antibodies at 4–6 months and administer boosters according to the result. This should be presented only as a hypothesis requiring prospective evaluation. Replace “hospitalization for severe disease” unless a validated definition of severe COVID-19 was applied.

Thank you for your valuable comment. We have revised the manuscript throughout to ensure that the findings are presented as associations rather than as independent prediction, vaccine effectiveness, or causation. We replaced “predicting the incidence and severity” with “examining associations with subsequent infection and hospitalization,” and references to prediction were changed to association where appropriate. We also removed the statement that binding antibodies “remained effective against Omicron” and removed references to the measured antibodies as neutralizing antibodies, as neutralization was not assessed in this study. The term “hospitalization for severe disease” was replaced with “hospitalization” where appropriate.

The previous recommendation to perform antibody testing at 4–6 months and administer booster doses according to antibody levels was removed. The Discussion was revised to present antibody-guided booster vaccination as a potential strategy requiring prospective evaluation. We now state that antibody testing may have a role in guiding booster vaccination strategies; however, the optimal timing of antibody testing and whether antibody levels can be used to guide the timing of booster doses remain uncertain and should be evaluated prospectively. The Conclusion was also revised accordingly. We now state that identifying transplant recipients with a low vaccine-induced antibody response may help guide the development of individualized immunization strategies, while emphasizing that further prospective studies are needed to determine whether antibody measurements could help guide individualized booster strategies and optimize protection in kidney transplant recipients.

3) The response to Reviewer 4 is substantially satisfactory, but similarity in age, sex, geographic/racial category, and transplant year does not eliminate selection bias. Clinical factors such as immunosuppressive regimen, renal function, comorbidities, healthcare utilization, or clinician reasons for ordering testing may still differ. The limitation should read: “The similarity of the measured baseline characteristics reduces concern regarding selection bias; however, residual selection bias related to unmeasured clinical characteristics or reasons for antibody testing cannot be excluded.”

Thank you for your valuable comment. We agree that similarity in the measured baseline characteristics does not eliminate the possibility of selection bias related to unmeasured factors. We have revised the Limitations section accordingly. The manuscript now states: “The similarity of the measured baseline characteristics reduces concern regarding selection bias; however, residual selection bias related to unmeasured clinical characteristics or reasons for antibody testing cannot be excluded.”

4) Define how COVID-19 infection was diagnosed—PCR, antigen testing, clinical diagnosis, or another criterion.

Thank you for your valuable comment. We have clarified the criteria used to diagnose COVID-19 infection in Section 2.3 (Data collection) of the Materials and Methods. All COVID-19 infections included in the study were PCR-confirmed. During the pre-Omicron period, diagnosis was based exclusively on PCR testing. During the Omicron-predominant period, rapid antigen testing was also used, but positive antigen test results required confirmation by PCR.

5) Explain how infections were attributed to Omicron, or revise the text to “infections occurring during the Omicron-predominant period.”

Thank you for your valuable comment. We agree that the infections cannot be definitively attributed to the Omicron variant. Therefore, we have revised the manuscript as suggested and replaced references to Omicron infections with “infections occurring during the Omicron-predominant period” where appropriate.

6) Include the informed-consent waiver in the submission-system ethics statement, consistent with the Methods.

Thank you for your valuable comment. We have updated the submission-system ethics statement to include the informed-consent waiver, consistent with the Methods section.

7) Correct language errors including “antibodyd,” “anit-SARS-CoV-2,” “A Poor response,” and inconsistent capitalization.

Thank you for your valuable comment. The identified language errors and inconsistencies in capitalization have been corrected in the revised manuscript.

8) Several errors need direct verification against the source dataset:

Lines 213–214: The manuscript says female sex was more common in the seronegative group “75% versus 62%.” Those are the reported male percentages and are also presented in the reverse order. Based on Table 2, female percentages appear to be 38% versus 25%.

Thank you for your valuable comment. The data were verified, and we confirmed that the previously reported percentages represented male rather than female participants. The percentages have been corrected in the revised manuscript to 38% versus 25%.

Lines 240–242: Diabetes is described as “69% versus 53%,” but Table 4 reports 69 patients (55%) versus 53 patients (42%).

Thank you for your valuable comment. The data were verified, and the percentages have been corrected in the revised manuscript to 55% versus 42%.

Lines 243–244: The text gives P = 0.02 for the interval between dose 2 and antibody testing, while Table 4 gives P = 0.002.

Thank you for your valuable comment. The data were verified, and the statistical analysis was repeated, confirming a P value of 0.002. This has been corrected in the revised manuscript.

Table 4: “Months from transplant” is reported as 86.8 versus 86.9 months with P = 0.02, while Table 5 reports no association after median categorization. The P value should be checked and the significant result discussed if correct.

Thank you for your valuable comment. We reviewed the statistical analysis and identified that an unpaired t-test had initially been used in Table 4 to compare months from transplant. Upon reviewing the distribution of the data, we found that the distribution of months from transplant was highly skewed, making an unpaired t-test inappropriate. We repeated the analysis using the Mann–Whitney U test, which is more appropriate for this non-normally distributed variable. The analysis yielded a P value of 0.51, and the P value in Table 4 has consequently been corrected from 0.02 to 0.51 in the revised manuscript.

Table 1 footnote: It incorrectly states that one-way ANOVA was used for categorical variables. Categorical comparisons should be identified as chi-square or Fisher’s exact tests. The authors should identify precisely whether ANOVA or Kruskal–Wallis testing was used for each continuous variable.

Thank you for your valuable comment. We have corrected the statistical methods reported in the Table 1 footnote. Categorical variables were compared using the chi-square test or Fisher’s exact test, as appropriate. For continuous variables, one-way ANOVA was used for variables with an approximately normal distribution, whereas the Kruskal–Wallis test was used for variables with a non-normal or markedly skewed distribution. Specifically, one-way ANOVA was used for age, while the Kruskal–Wallis test was used for SARS-CoV-2 antibody titer, days between the second vaccine dose and immunoassay, days between immunoassay and COVID-19 infection, and days between the third vaccine dose and COVID-19 infection. The Table 1 footnote has been revised, and the statistical methods have also been corrected accordingly in Section 2.4 (Statistical analysis) of the Materials and Methods.

Attachment Submitted filename: Response to academic editor (R4).docx

# Editor report: Decision Letter 4

**DOI:** 10.1371/journal.pone.0359640.r009

**Authors:** Stephen Walsh

17 Sep 2026

Assessment of Coronavirus Disease 2019 mRNA Vaccine Response in Kidney Transplant Recipients

PONE-D-24-28304R4

Dear Dr. Alkadi,

We’re pleased to inform you that your manuscript has been judged scientifically suitable for publication and will be formally accepted for publication once it meets all outstanding technical requirements.

Within one week, you’ll receive an e-mail detailing the required amendments. When these have been addressed, you’ll receive a formal acceptance letter and your manuscript will be scheduled for publication.

An invoice will be generated when your article is formally accepted. Please note, if your institution has a publishing partnership with PLOS and your article meets the relevant criteria, all or part of your publication costs will be covered. Please make sure your user information is up-to-date by logging into Editorial Manager at Editorial Manager® and clicking the ‘Update My Information' link at the top of the page. For questions related to billing, please contact billing support.

If your institution or institutions have a press office, please notify them about your upcoming paper to help maximize its impact. If they’ll be preparing press materials, please inform our press team as soon as possible -- no later than 48 hours after receiving the formal acceptance. Your manuscript will remain under strict press embargo until 2 pm Eastern Time on the date of publication. For more information, please contact onepress@plos.org.

Kind regards,

Stephen R. Walsh, MDCM

Academic Editor

PLOS One

# Editor report: Acceptance letter

**DOI:** 10.1371/journal.pone.0359640.r010

**Authors:** Stephen Walsh

PONE-D-24-28304R4

PLOS One

Dear Dr. Alkadi,

I'm pleased to inform you that your manuscript has been deemed suitable for publication in PLOS One. Congratulations! Your manuscript is now being handed over to our production team.

At this stage, our production department will prepare your paper for publication. This includes ensuring the following:

* All references, tables, and figures are properly cited

* All relevant supporting information is included in the manuscript submission,

* There are no issues that prevent the paper from being properly typeset

You will receive further instructions from the production team, including instructions on how to review your proof when it is ready. Please keep in mind that we are working through a large volume of accepted articles, so please give us a few days to review your paper and let you know the next and final steps.

Lastly, if your institution or institutions have a press office, please let them know about your upcoming paper now to help maximize its impact. If they'll be preparing press materials, please inform our press team within the next 48 hours. Your manuscript will remain under strict press embargo until 2 pm Eastern Time on the date of publication. For more information, please contact onepress@plos.org.

You will receive an invoice from PLOS for your publication fee after your manuscript has reached the completed accept phase. If you receive an email requesting payment before acceptance or for any other service, this may be a phishing scheme. Learn how to identify phishing emails and protect your accounts at https://explore.plos.org/phishing.

If we can help with anything else, please email us at customercare@plos.org.

Thank you for submitting your work to PLOS One and supporting open access.

Kind regards,

PLOS One Editorial Office Staff

on behalf of

Dr. Stephen R. Walsh

Academic Editor

PLOS One
